# Time-resolved proximity proteomics uncovers a membrane tension-sensitive caveolin-1 interactome at the rear of migrating cells

**DOI:** 10.7554/eLife.85601

**Published:** 2024-09-24

**Authors:** Eleanor Martin, Rossana Girardello, Gunnar Dittmar, Alexander Ludwig

**Affiliations:** 1 School of Biological Sciences, Nanyang Technological University Singapore Singapore; 2 NTU Institute of Structural Biology (NISB), Nanyang Technological University Singapore Singapore; 3 Proteomics of Cellular Signaling, Luxembourg Institute of Health Strassen Luxembourg; 4 Department of Life Sciences and Medicine, University of Luxembourg Esch-sur-Alzette Luxembourg; Institut de Biologie du Développement France; Fred Hutchinson Cancer Research Center United States

**Keywords:** caveolae, caveolin-1, proximity proteomics, APEX2, cell migration, membrane tension, mechanobiology, cavin, Human

## Abstract

Caveolae are small membrane pits with fundamental roles in mechanotransduction. Several studies have shown that caveolae flatten out in response to increased membrane tension, thereby acting as a mechanosensitive membrane reservoir that buffers acute mechanical stress. Caveolae have also been implicated in the control of RhoA/ROCK-mediated actomyosin contractility at the rear of migrating cells. However, how membrane tension controls the organisation of caveolae and their role in mechanotransduction remains unclear. To address this, we systematically quantified protein–protein interactions of caveolin-1 in migrating RPE1 cells at steady state and in response to an acute increase in membrane tension using biotin-based proximity labelling and quantitative mass spectrometry. Our data show that caveolae are highly enriched at the rear of migrating RPE1 cells and that membrane tension rapidly and reversibly disrupts the caveolar protein coat. Membrane tension also detaches caveolin-1 from focal adhesion proteins and several mechanosensitive regulators of cortical actin including filamins and cortactin. In addition, we present evidence that ROCK and the RhoGAP ARHGAP29 associate with caveolin-1 in a manner dependent on membrane tension, with ARHGAP29 influencing caveolin-1 Y14 phosphorylation, caveolae rear localisation, and RPE1 cell migration. Taken together, our work uncovers a membrane tension-sensitive coupling between caveolae and the rear-localised F-actin cytoskeleton. This provides a framework for dissecting the molecular mechanisms underlying caveolae-regulated mechanotransduction pathways.

## Introduction

Caveolae are an abundant feature of the plasma membrane of almost all vertebrate cells. They are composed of the scaffolding proteins caveolin-1 (Cav1) and caveolin-2 (Cav2), which are embedded in the inner leaflet of the plasma membrane, and the cavin protein family, which form a loose peripheral caveolar membrane coat. There are four cavins in humans (cavin1/PTRF, cavin2/SDPR, cavin3/SRBC, and cavin4/MURC). Cav1 and cavin1 are essential for caveolae formation, whereas cavins 2–4 regulate caveolae dynamics and functions in a cell and tissue-specific fashion (Kovtun et al., 2015; Parton et al., 2020; Shvets et al., 2014). A third caveolin protein (Cav3) and cavin4 are expressed exclusively in muscle cells. In addition, the ATPase EHD2 and the F-BAR domain containing protein Pacsin2 are found at caveolae. While caveolins and cavins assemble into an oligomeric membrane coat that shapes the caveolar bulb (Gambin et al., 2014; Kovtun et al., 2015; Kovtun et al., 2014; Ludwig et al., 2013; Ludwig et al., 2016; Stoeber et al., 2016), EHD2 and Pacsin2 are localised to the caveolar neck and link caveolae to actin filaments to stabilise caveolae at the plasma membrane and to restrict their mobility (Hansen et al., 2011; Ludwig et al., 2013; Morén et al., 2012; Senju et al., 2011; Stoeber et al., 2012).

Caveolae are extraordinarily dynamic in nature and have been implicated in diverse biological processes (Cheng and Nichols, 2016; Parton and Simons, 2007). Caveolae are abundant in cells that are exposed to tensile forces or shear stress, such as endothelial cells, epithelial cells, and muscle cells, and protect such cells from mechanical stress (Chai et al., 2013; Cheng et al., 2015; Dewulf et al., 2019; Garcia et al., 2017; Lim et al., 2017; Lo et al., 2015; Lu et al., 2017). There is evidence that caveolae disassemble (Sinha et al., 2011) or flatten out (Matthaeus et al., 2022) in response to an increase in membrane tension, and may similarly respond to other stresses such as osmotic stress (Guo et al., 2015), oxidative stress (Mougeolle et al., 2015), and UV irradiation (McMahon et al., 2019). Tension-induced flattening was proposed to be mediated (or accompanied) by the dissociation of the peripheral cavin coat and EHD2 from membrane-embedded caveolin oligomers (Sinha et al., 2011; Yeow et al., 2017). Cavin complexes and EHD2 released into the cytosol can subsequently translocate into the nucleus to regulate gene transcription (McMahon et al., 2021; McMahon et al., 2019; Torrino et al., 2018). This suggests that caveolae act as a dynamic mechanosensitive membrane reservoir that senses changes in membrane tension and transmits such inputs to downstream signalling (Del Pozo et al., 2021; Echarri and Del Pozo, 2015; Golani et al., 2019; Nassoy and Lamaze, 2012; Parton and del Pozo, 2013).

Large numbers of caveolae are also found at the rear of migrating cells (Hetmanski et al., 2019; Ludwig et al., 2013), and several studies indicate an intricate relationship between caveolae-mediated mechanosensing and RhoA-mediated cell rear retraction (Grande-García and del Pozo, 2008; Grande-García et al., 2007; Hetmanski et al., 2019). Caveolae formation at the cell rear is promoted by low membrane tension and is dependent upon RhoA/ROCK1 signalling, the Rho guanidine nucleotide exchange factor (GEF) Ect2, and the RhoA effector protein and serine-threonine kinase PKN2 (Hetmanski et al., 2019). Caveolae, in turn, enhance RhoA/ROCK1 signalling, leading to F-actin alignment, actomyosin contractility, and rapid rear retraction. Loss of caveolae, increased membrane tension, or inhibition of RhoA signalling all break this positive feedback loop, inhibiting rear retraction and impeding cell migration (Hetmanski et al., 2019; Hetmanski et al., 2021). This indicates a central role for caveolae in coupling changes in membrane tension to the control of actomyosin contractility at the rear of migrating cells. However, how membrane tension controls caveolae assembly and disassembly and caveolae-mediated signalling at the cell rear has not been analysed in a quantitative and objective manner.

Here we used live-cell imaging and electron tomography to study the dynamics and ultrastructure of caveolae at the rear of migrating RPE1 cells. We then employed proximity biotinylation with the peroxidase APEX2 and quantitative proteomics to define a Cav1-associated interactome in RPE1 cells at iso-osmotic conditions and in response to an acute increase in membrane tension elicited by hypo-osmotic shock. Our data demonstrate that membrane tension rapidly and reversibly disassembles the caveolae structure and abolishes the linkage between caveolae and the cortical actin cytoskeleton. In addition, we identify a number of potentially novel regulators and effectors of caveolae and present evidence that one of them, the Rho GTPase activating protein (GAP) ARHGAP29, controls caveolae rear localisation and RPE1 cell migration.

## Results

### Caveolae are abundant at and stably associated with the rear of RPE1 cells

**Figure 1.**
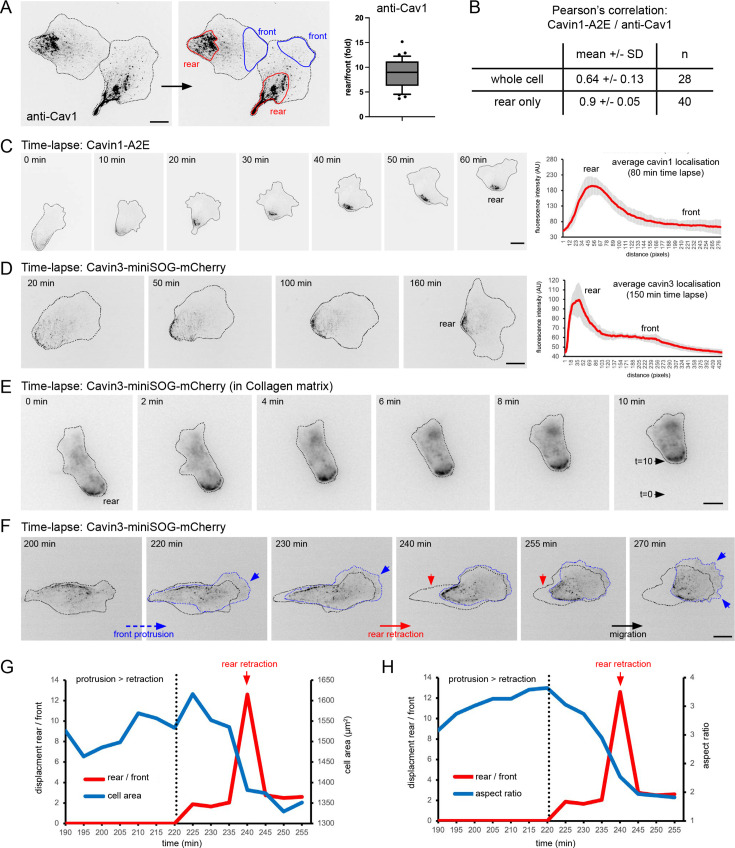
Dynamics of caveolae at the rear of migrating RPE1 cells. (**A**) RPE1 cells were fixed and stained with anti-Cav1 antibodies. The rear localisation index (fold enrichment at the rear) was determined by measuring the Cav1 intensity at the cell front and the cell rear (n = 36 cells). Scale bar 10 µm. (**B**) Pearson’s correlation analysis of fixed RPE1 cells stably expressing cavin1-A2E stained with anti-Cav1 antibodies. (**C**) Time-lapse images of RPE1 cells stably transfected with Cavin1-A2E migrating on a glass coverslip. The front-rear distribution of Cavin1-A2E in each frame of the movie was measured using line scans. The mean localisation was plotted. Error bars indicate SEM. Scale bar 10 µm. (**D**) Time-lapse images of RPE1 cells stably transfected with Cavin3-miniSOG-mCherry migrating on a glass coverslip. The front-rear distribution of Cavin3 in each frame of the movie was measured using line scans. The mean localisation was plotted. Error bars indicate SEM. Scale bar 10µm. (**E**) Time-lapse images of RPE1 cells stably transfected with cavin3-miniSOG-mCherry migrating on in a collagen matrix. Scale bar 5 µm. (**F**) Time-lapse images of RPE1 cells stably transfected with cavin3-miniSOG-mCherry showing a sudden rear retraction. Scale bar 10µm. (**G**) Temporal relationship between front protrusion, rear retraction, and cell area of the cell shown in (**F**). (**G**) Temporal relationship between front protrusion, rear retraction, and aspect ratio (cell shape) of the cell shown in (**F**).

We previously demonstrated that caveolae are enriched at the rear of hTERT-RPE1 cells (henceforth referred to as RPE1 cells) (Ludwig et al., 2013). Quantification of endogenous Cav1 fluorescence intensity in fixed RPE1 cells (visualised with anti-Cav1 antibodies) revealed an approximately eightfold enrichment at the cell rear compared to the cell front (Figure 1A). Such a polarised rear distribution of Cav1 is observed in ~70% of RPE1 cells sparsely grown on fibronectin-coated glass. To study the dynamics of caveolae in migrating RPE1 cells, we generated cell lines stably expressing fluorescently tagged cavin1 or cavin3 fusion proteins. Cavin1-APEX2-EGFP (Cavin1-A2E) was concentrated at the cell rear and colocalised with endogenous Cav1, as expected (Figure 1B, Figure 1—figure supplement 1A and B). Time-lapse microscopy and quantitative tracking of caveolae localisation in RPE1 cells migrating on a fibronectin-coated glass surface showed that both cavin1 and cavin3 fusion proteins were stably associated with the cell rear for several hours (Figure 1C and D, Figure 1—videos 1–3, Figure 1—figure supplement 1). Moreover, caveolae were associated with the rear of RPE1 cells migrating in a 3D collagen matrix (Figure 1E, Figure 1—video 4). Persistently migrating RPE1 cells showed relatively uniform protrusion and retraction speeds, resulting in an average translocation rate of ~0.4 µm/min. This is consistent with a previous report (Vaidžiulytė et al., 2022) and comparable to cell migration speeds of MEFs and MDCK cells and A2780 cells migrating in durotactic gradients (Hetmanski et al., 2019; Sitarska and Diz-Muñoz, 2020). When cells re-oriented their front-rear axis, this uniform protrusion/retraction cycle was transiently interrupted. The cells ceased to move and waves of new protrusions formed to establish a new leading edge. Interestingly, during this protrusion phase the rear remained relatively static, leading to a slow but persistent increase in cell length (aspect ratio) and cell area. At a certain point, the rear suddenly retracted, causing instantaneous and large cell rear displacements and an apparent accumulation of caveolae at the rear (Figure 1F–H, Figure 1—figure supplement 1D and F). Complete reversals of the front-rear axis were also frequently observed. In such cases, the polarised distribution of caveolae was transiently lost but was rapidly re-established after a new front-rear axis had been established (Figure 1—video 7). Similarly, caveolae rear localisation was rapidly re-established after cell division (Figure 1—video 8). Our live-cell imaging data further show that when cells polarise spontaneously, caveolae become aligned along the long cell axis in a filament-like pattern and eventually accumulate at the rear membrane as the rear retracts and the polarity axis is established (Figure 1—figure supplement 2, Figure 1—videos 9 and 10). Together this indicates that caveolae are stably associated with the RPE1 cell rear and suggests that rear retraction promotes the accumulation of caveolae at the trailing edge.

**Figure 1—figure supplement 1.**
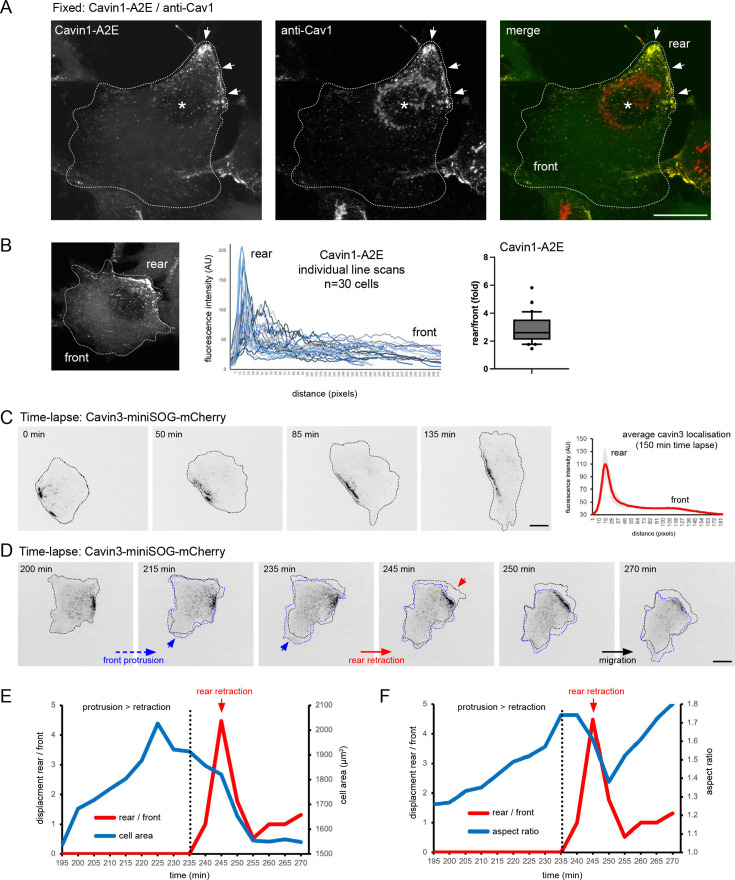
Characterisation of caveolae rear localisation in RPE1 cells. (**A**) RPE1 cells stably transfected with cavin1-A2E were fixed and stained with anti-Cav1 antibody. Note the colocalisation of Cav1 and cavin1 at the rear (arrows). The Golgi apparatus (*) is stained with anti-Cav1 antibody but not the cavin1 fusion protein. (**B**) Quantification of cavin1 rear localisation in fixed cells. A representative confocal image of cavin1-A2E stably expressed in RPE1 cells is shown. The front-rear distribution of cavin1-A2E was measured in 30 individual cells using line scans. In addition, the rear localisation index (fold enrichment at the rear) was determined by measuring the cavin1 intensity at the cell front and the cell rear (n = 30 cells). (**C**) Time-lapse images of RPE1 cells stably transfected with Cavin3-miniSOG-mCherry migrating on a glass coverslip. The front-rear distribution of Cavin3 in each frame of the movie was measured using line scans. The mean localisation was plotted. Error bars indicate SEM. (**D**) Time-lapse images of RPE1 cells stably transfected with cavin3-miniSOG-mCherry showing a sudden rear retraction. (**E**) Temporal relationship between front protrusion, rear retraction, and cell area of the cell shown in (**D**). (**F**) Temporal relationship between front protrusion, rear retraction, and aspect ratio (cell shape) of the cell shown in (**D**). Scale bars 10 µm.

**Figure 1—figure supplement 2.**
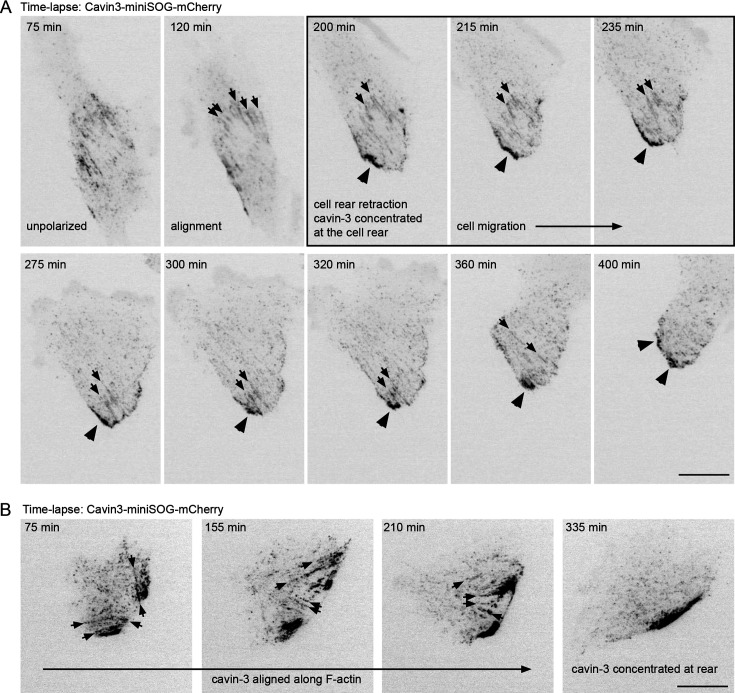
Caveolae become transiently aligned along the front/rear axis in migrating RPE1 cells. (**A**) Time-lapse images of RPE1 cells stably transfected with Cavin3-miniSOG-mCherry. The movie starts with an unpolarised cell state. Cavin3 becomes aligned in ‘stripes’ as the cell establishes a front and a rear (small arrows). A sudden rear retraction at 200 min is accompanied with an accumulation of cavin3 at the rear (large arrowheads). The cell then starts to migrate. (**B**) Time-lapse images showing the alignment of cavin3 in ‘filamentous stripes’ (small arrows) in a migrating RPE1 cell. Scale bars 10 µm.

**Figure 1—video 1.** Time-lapse microscopy of a persistently migrating RPE1 cell stably expressing cavin1-APEX2-EGFP (related to Figure 1C).

**Figure 1—video 2.** Time-lapse microscopy of a persistently migrating RPE1 cell stably expressing cavin3-miniSOG-mCherry (related to Figure 1D).

**Figure 1—video 3.** Time-lapse microscopy of a persistently migrating RPE1 cell stably expressing cavin3-miniSOG-mCherry (related to Figure 1—figure supplement 1C).

**Figure 1—video 4.** Time-lapse microscopy of an RPE1 cell stably expressing cavin3-miniSOG-mCherry persistently migrating in a 3D collagen matrix (related to Figure 1E).

**Figure 1—video 5.** Time-lapse microscopy of an RPE1 cell stably expressing cavin3-miniSOG-mCherry. Sudden rear retractions with large rear replacements can be seen between 145 and 150 min, between 235 and 240 min, and between 705 and 715 min of the movie (related to Figure 1F).

**Figure 1—video 6.** Time-lapse microscopy of an RPE1 cell stably expressing cavin3-miniSOG-mCherry. A sudden rear retraction can be seen between 235 and 250 min of the movie (related to Figure 1—figure supplement 1D).

**Figure 1—video 7.** Time-lapse microscopy of an RPE1 cell stably expressing cavin3-miniSOG-mCherry undergoing a complete reversal of front-rear polarity. The cell changes its front-rear axis between 140 and 200 min of the movie, which is accompanied by a reorganisation of cavin3.

**Figure 1—video 8.** Time-lapse microscopy of an RPE1 cell stably expressing cavin3-miniSOG-mCherry undergoing cell division. Abscission of the cytokinetic bridge can be observed at 175 min. Cavin3 rear localisation is evident and the cells start to migrate.

**Figure 1—video 9.** Time-lapse microscopy of an RPE1 cell stably expressing cavin3-miniSOG-mCherry undergoing front-rear polarisation. Caveolae become initially aligned along the long cell axis and progressively accumulate at the rear as the cell polarises and the rear retracts (160–250 min) (related to Figure 1—figure supplement 2A).

**Figure 1—video 10.** Time-lapse microscopy of an RPE1 cell stably expressing cavin3-miniSOG-mCherry. Note that cavin3 is aligned in ‘stripes’ or ‘filaments’ as the cell migrates (related to Figure 1—figure supplement 2B).

**Figure 2.**
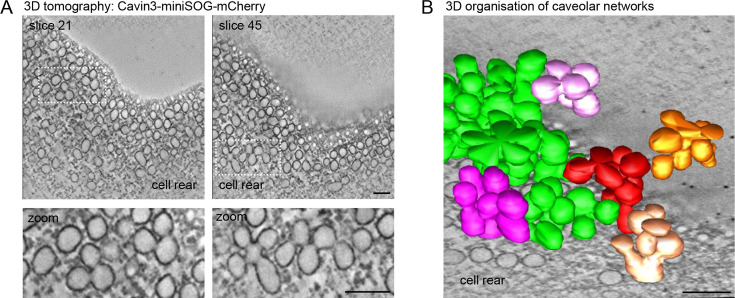
Electron tomography of caveolae at the RPE1 cell rear. (**A**) Electron tomography of caveolae and caveolar networks at the rear of RPE1 cells stably transfected with Cavin3-miniSOG-mCherry. Two slices of the tomogram are shown. Note that individual caveolae are interconnected. (**B**) 3D surface model of the tomogram shown in (**A**), showing interconnected clusters, or rosettes, of caveolae. Scale bar in (**A**) and (**B**) is 100 nm.

Next we used RPE1 cells stably expressing a cavin3-miniSOG-mCherry fusion protein to visualise the 3D architecture of caveolae at the cell rear through miniSOG labelling and 3D electron tomography (Ludwig et al., 2013; Shu et al., 2011). 3D tomography and surface rendering of the tomograms revealed a dense network of surface-connected caveolae and the presence of large interconnected caveolar clusters, most of which appeared to be continuous with the rear plasma membrane (Figure 2A and B, Figure 2—video 1). We concluded that caveolae are abundant at the rear of RPE1 cells, which hence provide an appropriate model cell type to study the caveolae-associated protein network in migrating cells.

**Figure 2—video 1.** Electron tomogram and surface rendering of caveolar clusters and networks at the rear of RPE1 cells stably expressing cavin3-miniSOG-mCherry. Caveolae were specifically labelled using miniSOG (related to Figure 2B).

### The caveolin-1 interactome is enriched in cortical actin regulators and is regulated by membrane tension

**Figure 3.**
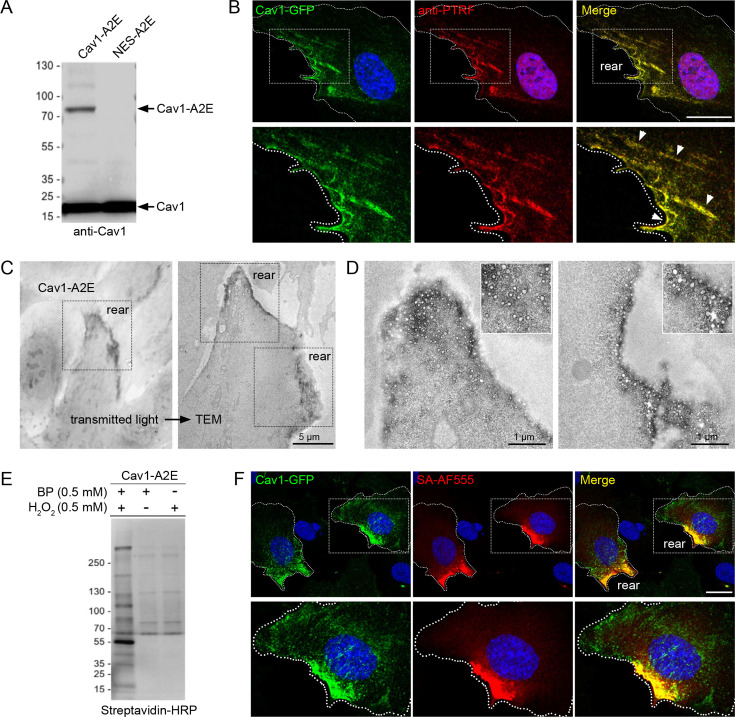
Proximity labelling of caveolae in RPE1 cells stably expressing Cav1-APEX2-EGFP. (**A**) Western blot of stable RPE1 cell lines expressing either Cav1-A2E or NES-A2E fusion proteins. The membrane was probed with anti-Cav1 antibodies. (**B**) Confocal fluorescence microscopy of Cav1-A2E RPE1 cells stained with anti-PTRF/cavin1 antibodies. Boxed regions are magnified in the bottom panel. Nuclei were counterstained with DAPI. Scale bar 10 µm. (**C**) Transmitted light image of Cav1-A2E RPE1 cells after APEX2 labelling and plastic embedding (left) and correlative TEM image of the same cell (right). (**D**) Representative TEM micrographs of serial TEM sections of the cell shown in (**C**) (boxed regions). Note the specific labelling and abundance of caveolae at the cell rear. (**E**) Cav1-A2E-expressing cells were incubated in the absence or presence of biotin phenol and/or hydrogen peroxide. Cell lysates were separated by SDS-PAGE, blotted onto PVDF membrane, and probed with streptavidin-HRP. (**F**) Confocal microscopy of Cav1-A2E RPE1 cells after proximity labelling with APEX2. Cells were fixed and stained with fluorescent streptavidin (Alexa Fluor 555) to visualise biotinylated proteins. Nuclei were counterstained with DAPI. Scale bar 10 µm. Figure 3—source data 1.Original western blots.

Having established that caveolae are enriched at the cell rear, we aimed to identify proteins associated with caveolae in migrating cells and ask how such a protein network would respond to an acute increase in membrane tension. Several cytoplasmic effectors of cavins have previously been identified using BioID-mediated proximity proteomics (McMahon et al., 2021; McMahon et al., 2019; Mendoza-Topaz et al., 2018). However, BioID requires long labelling times (16–24 hr) and therefore is not suitable to dissect rapid changes in the caveolae-associated protein network that are likely to occur as cells respond to acute mechanical stress. To address this we employed a proximity biotinylation approach using the APEX2 peroxidase (Hung et al., 2016; Lam et al., 2015). A stable RPE1 cell line expressing a Cav1-APEX2-EGFP (Cav1-A2E) fusion protein was generated. We have previously shown that expression of this construct rescues caveolae formation in mouse embryonic fibroblasts from *CAV1* -/- mice, indicating that this fusion protein is functional (Ludwig et al., 2017; Ludwig et al., 2016). Cav1-A2E in RPE1 cells was expressed at levels comparable to or lower than that of endogenous Cav1 (Figure 3A), colocalised with PTRF/cavin1 at the cell rear (Figure 3B, Figure 3—figure supplement 1A), and was stably associated with the rear in migrating cells (Figure 3—figure supplement 1B, Figure 3—video 1). Moreover, transmission electron microscopy showed that the Cav1-A2E fusion protein was efficiently incorporated into caveolae (Figure 3C and D).

**Figure 3—figure supplement 1.**
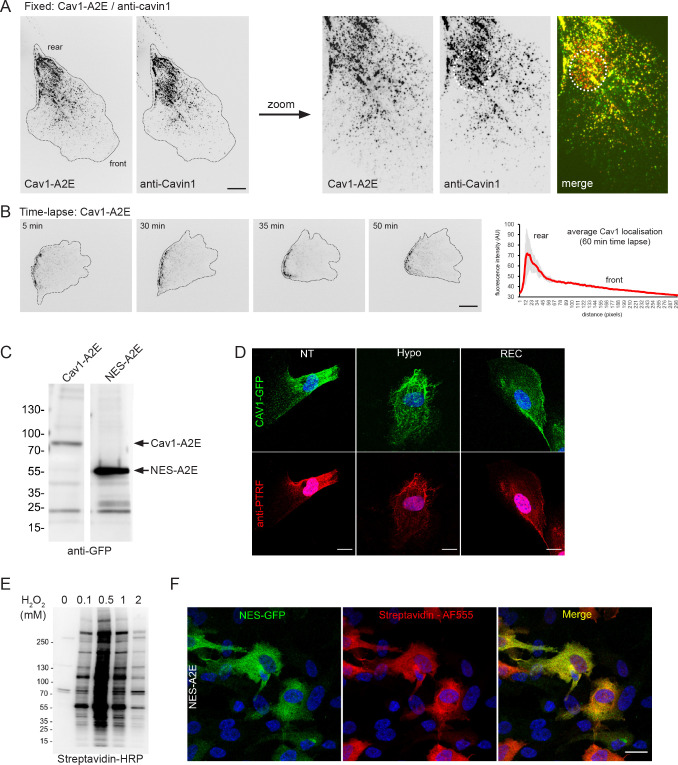
Characterisation of the Cav1-A2E RPE1 cell lines. (**A**) RPE1 cells stably transfected with Cav1-A2E were fixed and stained with anti-cavin1 antibodies. Note the pronounced colocalisation and the enrichment of Cav1-A2E and cavin1 at the cell rear. Note that cavin1, but not Cav1, is found in the nucleus (circle). Scale bar 10 µm. (**B**) Time-lapse images of RPE1 cells stably transfected with Cav1-A2E migrating on a glass coverslip. The front-rear distribution of Cav1-A2E in each frame of the movie was measured using line scans. The mean localisation was plotted. Error bars indicate SEM. Scale bar 10 µm. (**C**) Western blot analysis of protein lysates from RPE1 cells stably expressing Cav1-A2E or NES-A2E fusion proteins. The membrane was probed with anti-GFP antibodies. (**D**) Representative immunofluorescence stainings of Cav1-A2E RPE1 cells at isotonic (NT) and hypotonic (HYPO) conditions, and after recovery from hypo-osmotic shock (REC). Cells were co-stained with anti-PTRF/cavin1 antibodies. Scale bar 20 µm. (**E**) H_2_O_2_ ramp of NES-A2E-expressing RPE1 cells pre-incubated for 30 min with 0.5 mM biotin phenol. Increasing concentrations of H_2_O_2_ were used during the biotinylation reaction to determine the optimal H_2_O_2_ concentration for further experiments. (**F**) Confocal microscopy of NES-A2E cells after proximity labelling with APEX2. Cells were fixed and stained with fluorescent streptavidin (Alexa Fluor 555) to visualise biotinylated proteins. Nuclei were counterstained with DAPI. Scale bar 20 µm. Figure 3—figure supplement 1—source data 1.Original western blots.

**Figure 3—video 1.** Time-lapse microscopy of a persistently migrating RPE1 cell stably expressing Cav1-APEX2-EGFP (related to Figure 3—figure supplement 1B).

Next we ascertained that proximity biotinylation in the Cav1-A2E cell line is specific and spatially restricted. Streptavidin-HRP blotting of Cav1-A2E RPE1 cell lysates showed that the biotinylation reaction was dependent upon the presence of biotin phenol and a brief (1 min) exposure to hydrogen peroxide (Figure 3E). In addition, fluorescent streptavidin labelling showed that the biotinylation reaction was restricted to the cell rear and colocalised with Cav1-A2E (Figure 3F). We also generated a control RPE1 cell line expressing APEX2-EGFP fused to a nuclear export signal (NES-A2E). In this cell line biotinylated proteins were diffusely localised in the cytoplasm, as shown previously (Figure 3—figure supplement 1C–F; Tan et al., 2020a).

**Figure 4.**
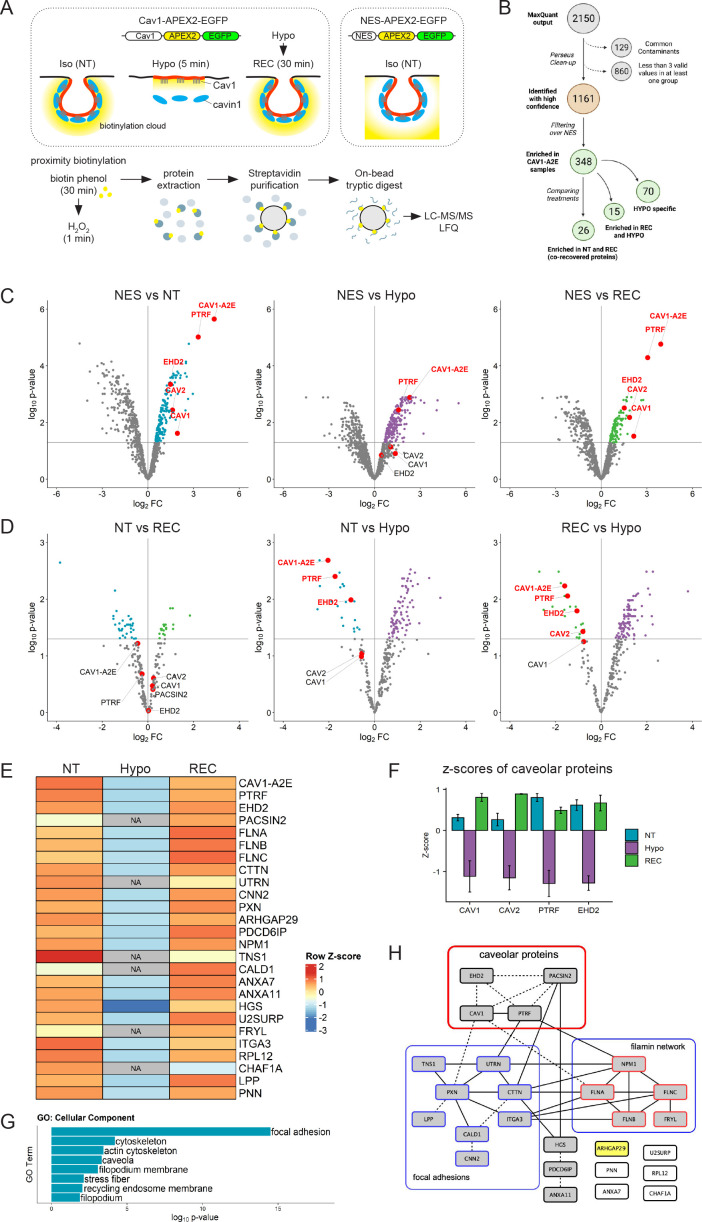
The caveolin-1 interactome is enriched in cortical actin regulators and focal adhesion proteins and is highly sensitive to membrane tension. (**A**) Experimental set-up used for APEX2-mediated proximity biotinylation and LC-MS/MS. After proximity labelling, cells were lysed and biotinylated proteins were captured using magnetic streptavidin beads. Purified proteins were on bead-digested with trypsin and the recovered peptides analysed by LC-MS/MS. Three individual replicates were analysed. (**B**) Diagram summarising the LC-MS/MS analysis and the total number of proteins significantly enriched in each treatment compared to the NES control. Only proteins identified in all three replicates in at least one of the three samples were considered for further analysis. Label-free quantification (LFQ) was used to define specific Cav1 interactomes and to determine quantitative changes upon hypo-osmotic shock and recovery. (**C**) Volcano plots showing proteins significantly enriched in NT, HYPO, and REC samples compared to the NES control sample. Only proteins with a p-value <0.05 and a logFC > 0 were considered for further analysis. (**D**) Volcano plots comparing the NT and REC, NT and HYPO, and REC and HYPO samples. Cav1-A2E fusion protein and the core caveolar proteins are highlighted. (**E**) Heatmap of z-scored LFQ quantified protein groups differentially enriched across the treatments. NA indicates not identified. See Figure 3—figure supplement 1 for a complete analysis. (**F**) Z-scores of caveolar proteins under NT, HYPO and REC conditions. (**G**) Gene Ontology (GO) Cellular Component analysis for proteins enriched under NT and REC conditions (see **E**). (**H**) PPI network of the Cav1 proximity proteome. Only proteins significantly enriched in both the NT and REC samples were considered (i.e., 26 co-recovered proteins). Solid lines indicate interactions retrieved from databases (BioGRID, Reactome, BioCarta), dotted lines indicate interactions retrieved from a hand-curated literature search (see Supplementary file 2).

To generate Cav1 proximity proteomes at resting conditions and upon an acute increase in membrane tension, we used a hypo-osmotic shock assay (Sinha et al., 2011; Figure 4A). Cav1-A2E-expressing cells were either grown in isotonic medium and left untreated (non-treated; NT), exposed to a brief 5 min hypo-osmotic shock (HYPO), or exposed to hypo-osmotic shock and allowed to recover in iso-osmotic medium for 30 min (REC). NES-A2E cells grown in iso-osmotic medium (NT) were used as a reference (or ‘spatial ruler’) to subtract abundant cytoplasmic proteins and non-specific bystanders. After ratiometric filtering over the NES sample, we identified a total of 348 unique proteins that were significantly enriched in the three Cav1 proximity proteomes (see Supplementary file 1 for the mass spectrometry data). In total, 70 proteins were exclusive to the HYPO sample, 15 proteins were enriched specifically in the HYPO and REC samples, and 26 proteins were enriched specifically in the NT and REC samples (Figure 4B, Figure 4—figure supplement 1). Importantly, Cav1-A2E and all major caveolar proteins (Cav1, Cav2, PTRF/cavin1, and EHD2; note that cavin2 and cavin3 are not expressed in RPE1 cells) were specifically enriched in the iso-osmotic control (NT) and recovered (REC) samples. On the contrary, under hypo-osmotic (HYPO) conditions, Cav1, Cav2, and EHD2 were no longer significantly enriched with Cav1-A2E (Figure 4C), suggesting that high membrane tension had caused the dissociation of these proteins from Cav1-A2E. Indeed, Cav1-A2E, PTRF/cavin1, and EHD2 were all specifically enriched in the NT and REC samples when directly compared to the HYPO sample (Figure 4D). By contrast, no significant differences in the abundance of caveolar core components were observed between NT and REC samples, suggesting that caveolae formation is fully restored within 30 min of recovery from a hypo-osmotic shock. We concluded that proteins identified specifically in the NT and REC samples are associated with Cav1 in a membrane-tension-dependent manner and therefore constitute a reliable Cav1 proximity proteome under iso-osmotic conditions.

**Figure 4—figure supplement 1.**
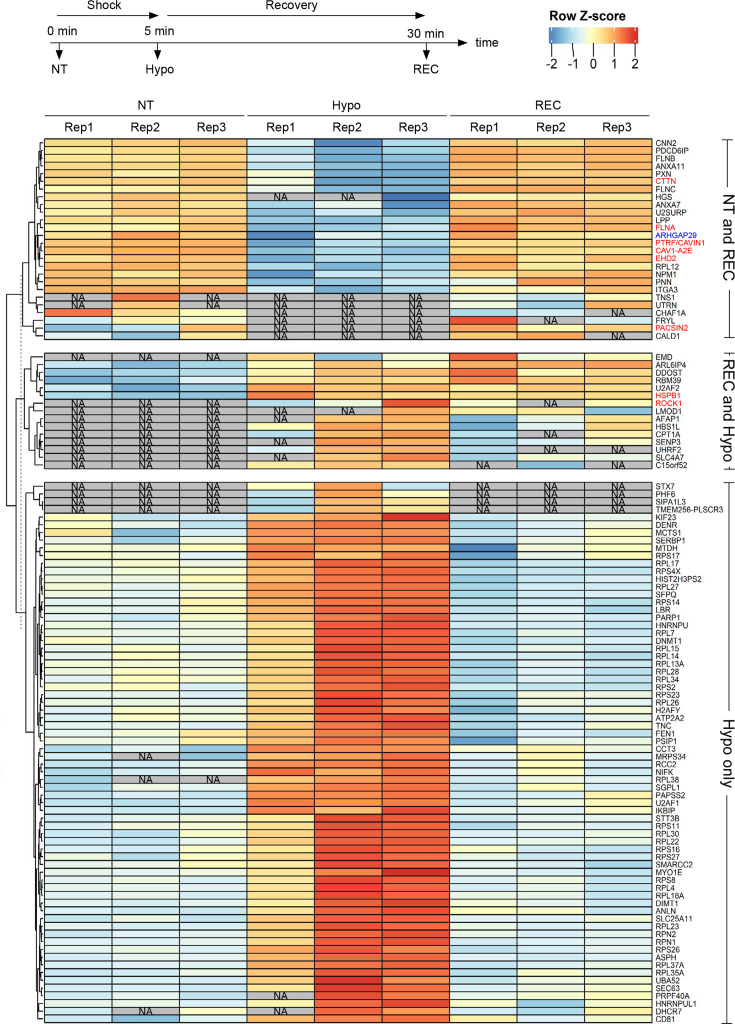
Quantitative proximity proteomics of Cav1-APEX2-EGFP in response to hypo-osmotic shock. Heatmap of 111 z-scored LFQ quantified protein groups differentially enriched across the three treatments (non-treated [NT, control], hypo-osmotic shock [HYPO], and recovery from hypo-osmotic shock [REC]). All three replicates are shown. NA indicates not identified.

Quantification of the MS data and GO annotation analyses revealed that the Cav1 proximity proteome was enriched in components of the cortical actin cytoskeleton and integrin adhesions, including filamins (FLNA, FLNB, FLNC), utrophin (UTRN), tensin-1 (TNS1), cortactin (CTTN), and paxillin (PXN) (Figure 4E and G, Figure 4—figure supplement 1). Of note, all caveolar proteins showed negative Z-scores (i.e., were under-represented) under hypo-osmotic conditions (Figure 4F). Some proteins such as Pacsin2, utrophin, and tensin-1 were entirely absent from the Cav1 proximity proteome obtained in hypo-osmotic medium (Figure 4E; indicated as NA). A comprehensive database and literature review further identified several direct and indirect interactions between caveolar proteins, filamins and focal adhesion components (Figure 3H). These data support previous work demonstrating a role for caveolae in focal adhesion turnover and integrin-mediated mechanotransduction (Joshi et al., 2008; Lolo et al., 2022; Meng et al., 2017; Nethe and Hordijk, 2011). Moreover, the F-actin crosslinking protein filamin-A and utrophin are well-known mechanotransducers (Rajaganapathy et al., 2019; Stossel et al., 2001) and have been shown to regulate caveolae trafficking and dynamics, possibly by linking caveolae to the actin cytoskeleton via direct or indirect interactions with Cav1 or PTRF/cavin1 (Kanai et al., 2014; Muriel et al., 2011; Palma-Flores et al., 2014; Stahlhut and van Deurs, 2000; Sverdlov et al., 2009). Filamins and utrophin, as well as caldesmon-1 (CALD1) and calponin-2 (CNN2), have also all been implicated in the control of cell migration (Baldassarre et al., 2009; Hossain et al., 2016; Li et al., 2013; Liu and Jin, 2016; Mayanagi and Sobue, 2011; Sobue and Sellers, 1991; Ulmer et al., 2013). We concluded that time-resolved proximity labelling with APEX2 uncovered a dynamic Cav1 interactome enriched in mechanosensitive cortical actin regulators, which is highly sensitive to acute changes in membrane tension.

**Figure 5.**
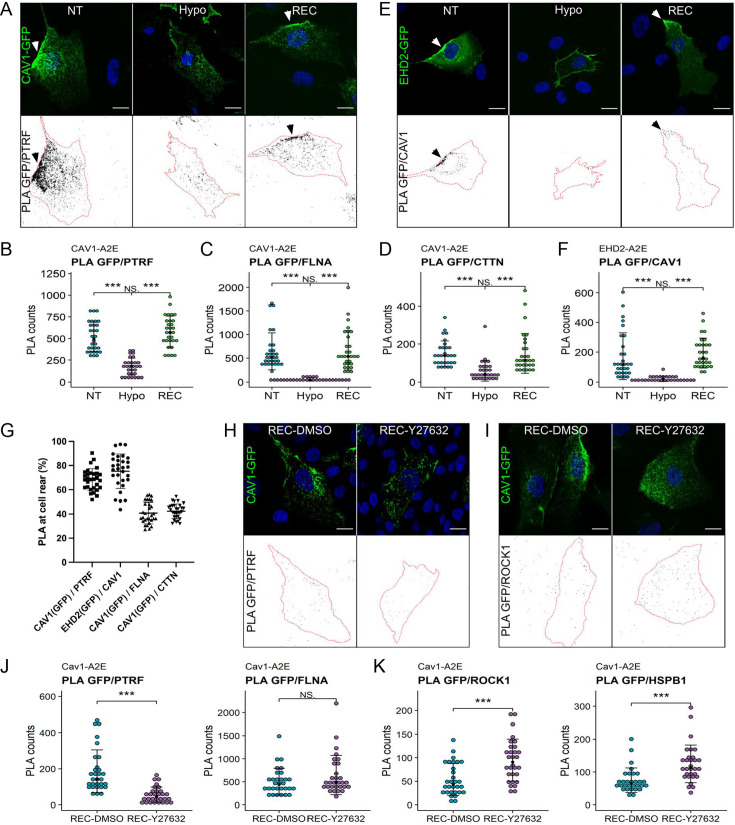
Proximity ligation assays (PLA) reveal dynamic interactions between caveolae and the cortical actin cytoskeleton during changes in membrane tension. (**A–D**) PLA in the Cav1-A2E RPE1 cell line using anti-GFP and anti-PTRF/cavin1 (**A, B**), anti-FLNA (**C**), or anti-CTTN (**D**) antibodies as indicated. (**E, F**) PLA in RPE1 cells transfected with EHD2-A2E using anti-GFP and anti-Cav1 antibodies. (**G**) Quantification of rear-localised PLA signals based on data shown in panels (**A–F**). (**H–K**) Effect of ROCK inhibition on PLA in the Cav1-A2E RPE1 cell line using the indicated combinations of antibodies. Cells were incubated for 5 min in hypo-osmotic medium containing 10 µM Y-27632 or DMSO (control) and then transferred to isotonic medium for 30 min in the absence (DMSO) or presence of Y-27632. The graphs in (**B, C, D, F, J, K**) show the quantifications of the PLA signals per cell. 30 cells from three independent experiments were quantified for each treatment. The number of PLA dots per cell is presented with the mean values ± SD. The bottom panels in (**A, E, H, I**) show the PLA signal masks used for counting. Statistical significance was calculated using a Wilcoxon test. ns = p>0.05, ***p≤0.001. All scale bars 20 µm.

To validate our proteomics data, we quantified the spatial proximity between Cav1 and some of the identified proteins using proximity ligation assays (PLA) (Figure 5A–F, Figure 5—figure supplement 1). In agreement with our proteomics data, under iso-osmotic conditions we observed robust PLA signals for Cav1-A2E and PTRF/cavin1, Cav1-A2E and filamin-A, and Cav1-A2E and cortactin (Figure 5A–D). Moreover, robust signals were observed for endogenous Cav1 and an APEX2-EGFP fusion protein of EHD2 (EHD2-A2E) (Figure 5E and F). As expected, 70–80% of the PLA signals for Cav1-A2E and PTRF/cavin1 and anti-Cav1 and EHD2-A2E localised to the cell rear, whilst rear-localised PLA signals for Cav1-A2E and filamin-A and Cav1-A2E and cortactin were significantly lower (~40%) (Figure 5G). This is expected as filamin-A and cortactin were present but not enriched at the cell rear (Figure 5—figure supplement 1). Importantly, hypo-osmotic shock drastically reduced the PLA signals in all of the above cases, whereas cells that had recovered from the hypo-osmotic shock showed PLA signals indistinguishable to that of control cells. Taken together our data indicate that a brief hypo-osmotic shock causes an instantaneous but reversible disassembly of caveolae, as shown previously (Sinha et al., 2011; Torrino et al., 2018; Yeow et al., 2017). The data further imply that membrane tension regulates the linkage between caveolae and the cortical actin cytoskeleton and that caveolae assembly/disassembly is spatio-temporally coordinated with the reorganisation of the rear-localised actin cortex.

**Figure 5—figure supplement 1.**
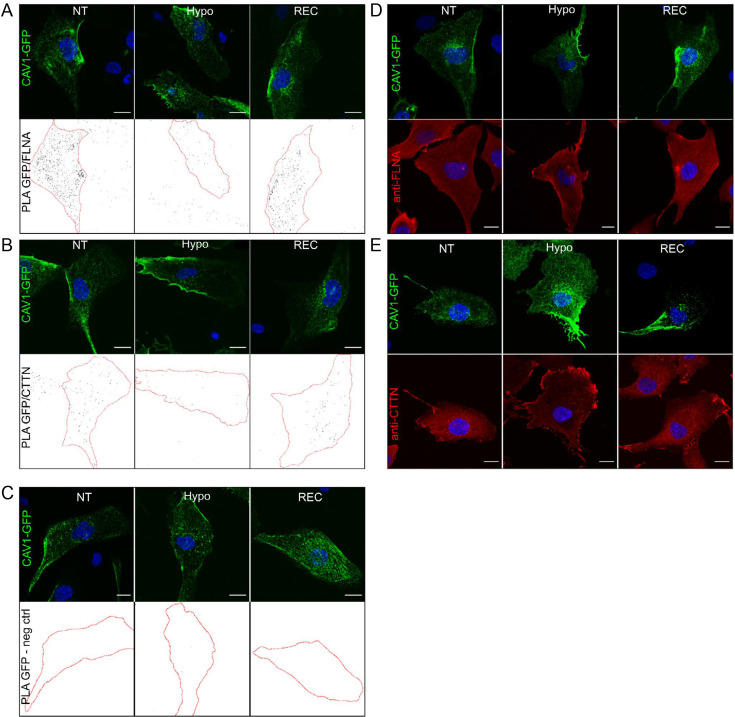
Proximity ligation assays (PLA) in Cav1-A2E RPE1 cells using CTTN and FLNA antibodies. (**A, B**) Representative images of PLA on Cav1-A2E-expressing RPE1 cells using anti-GFP antibodies and anti-FLNA (**A**) or anti-CTTN antibodies (**B**). (**C**) Control PLA of Cav1-A2E cells. Samples were probed only with anti-GFP primary antibody and both PLA probes. GFP and DAPI signals used to determine cell boundaries are shown in the upper panels in (**A–C**), while the bottom panels show the PLA signal masks used for counting. (**D, E**) Representative immunofluorescence images of fixed Cav1-A2E cells co-stained with anti-FLNA (**D**) or anti-CTTN antibodies (**E**). Scale bars 20 µm.

**Figure 5—figure supplement 2.**
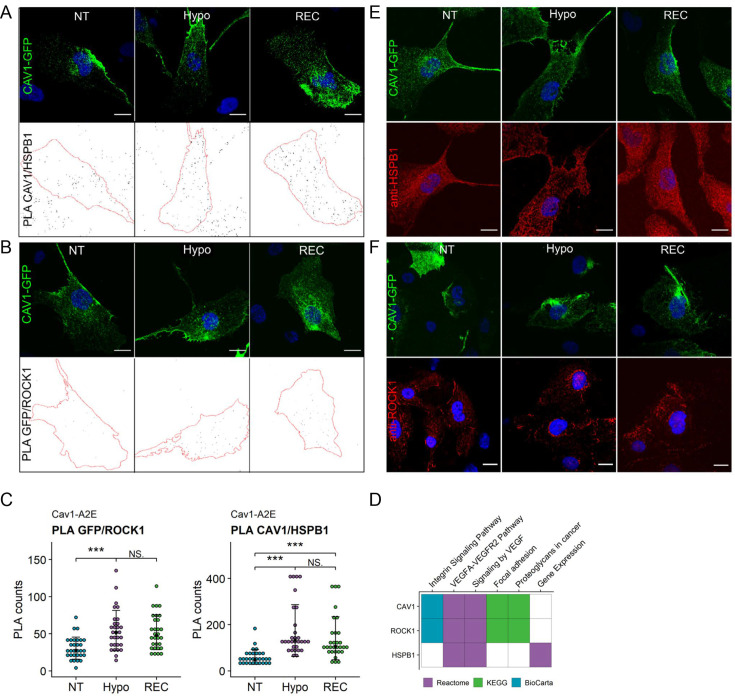
Proximity ligation assays (PLA) in Cav1-A2E RPE1 cells using ROCK1 and HSPB1 antibodies. (**A, B**) Representative images of PLA on Cav1-A2E-expressing RPE1 cells using anti-GFP antibodies and anti-HSPB1 (**A**) or anti-ROCK1 antibodies (**B**). GFP and DAPI signals used to determine cell boundaries are shown in the upper panels in (**A**) and (**B**), while the bottom panels show the PLA signal masks used for counting. Scale bars 20 µm. (**C**) PLA quantification of the data shown in (**A**) and (**B**). (**D**) Pathway enrichment analysis of proteins that are significantly enriched in the REC and HYPO samples compared to NT. (**E, F**) Representative immunofluorescence images of fixed Cav1-A2E cells co-stained with anti-HSPB1 (**E**) or anti-ROCK1 antibodies (**F**). Scale bars 20 µm.

### ROCK activity is required for caveolae reassembly upon hypo-osmotic shock

Interestingly, several proteins were specifically enriched with Cav1 during the recovery phase (i.e., in HYPO and REC samples) (Figure 4—figure supplement 1). Among these proteins were Rho-associated protein kinase 1 (ROCK1) and the heat shock protein HSPB1. Rho/ROCK1 signalling was previously shown to be required for caveolae localisation to the rear of migrating cells (Hetmanski et al., 2019) and HSPB1 is a stress-regulated actin binding protein implicated in mechanotransduction and cell migration (Clarke and Mearow, 2013; Collier et al., 2019; Hoffman et al., 2022). PLAs confirmed that the association of both HSPB1 and ROCK1 with Cav1 is significantly enhanced in cells exposed to hypo-osmotic shock and in cells that had recovered from the shock (Figure 5—figure supplement 2). In addition, pathway enrichment analysis suggested a functional link between Cav1, ROCK1, and HSPB1 (Figure 5—figure supplement 2D). To test whether ROCK1 activity, and hence actomyosin contractility, is required for caveolae reformation following hypo-osmotic shock, we performed PLAs in the absence or presence of the ROCK inhibitor Y-27632. ROCK inhibition blocked caveolae rear localisation (Figure 5H and I) and significantly reduced the PLA signal between Cav1-A2E and PTRF/cavin1, but did not affect the Cav1-A2E/filamin-A interaction (Figure 5J). Interestingly, PLA signals between Cav1-A2E and ROCK1 as well as between Cav1-A2E and HSPB1 were significantly increased upon ROCK inhibition (Figure 5K). This corroborates our proximity proteomics data and suggests that (a) ROCK1 and HSPB1 are recruited to caveolae (or the cell rear) at high membrane tension, (b) that ROCK activity is required for caveolae reassembly after hypo-osmotic shock, and (c) that ROCK activity weakens the protein’s association with caveolae (or the cell rear).

### ARHGAP29 controls caveolin-1 phosphorylation, caveolae rear localisation, and RPE1 cell migration

**Figure 6.**
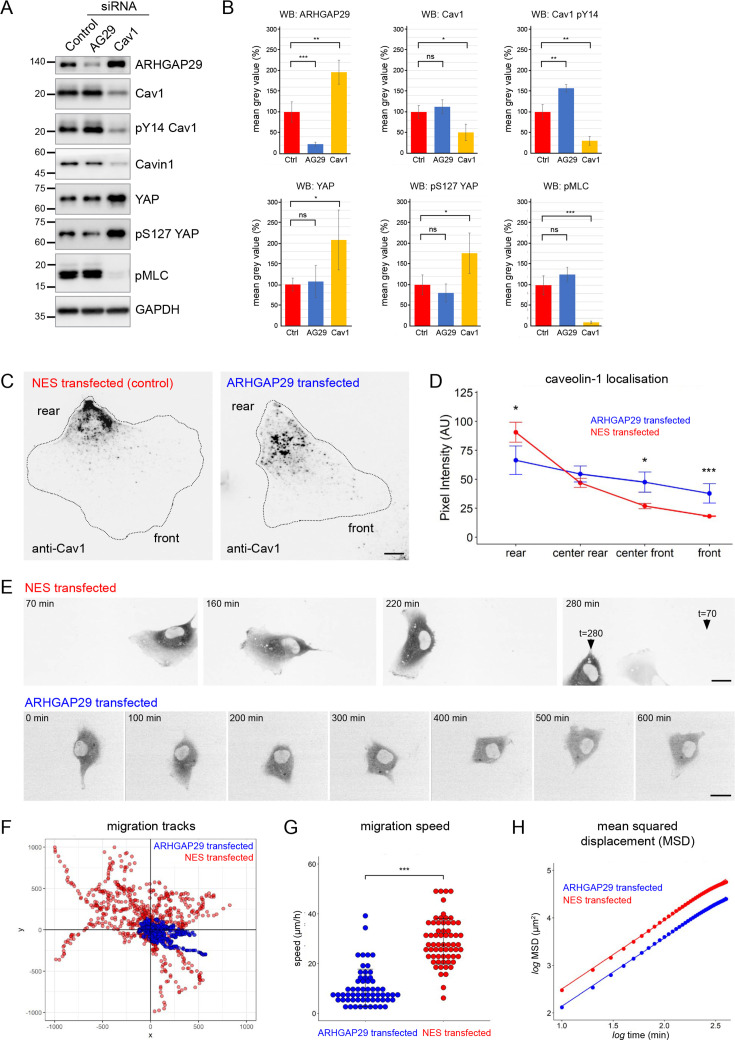
Evidence for a functional connection between caveolae and ARHGAP29. (**A**) RPE1 cells were transfected with siRNAs against ARHGAP29, caveolin-1, or non-targeting siRNAs and analysed by western blotting 48 hr post-transfection. (**B**) Quantification of western blot analysis shown in (**A**). Ratios normalised to the GAPDH loading control are displayed relative to the intensity of the control siRNA transfection for each protein indicated. Data represent the mean ± SD of 3–4 independent experiments. Statistical significance was calculated using an unpaired *t*-test. ns = p>0.05, *p≤0.05, **p≤0.01, ***p≤0.001. (**C**) RPE1 cells were transfected with A2E-ARHGAP29 or NES-A2E, fixed, and stained with anti-Cav1 antibodies. Scale bar 5 µm. (**D**) Quantification of Cav1 rear localisation based on data shown in (**C**). Error bars indicate mean ± SEM *p≤0.05, ***p≤0.001, Wilcoxon test (n = 18 cells per condition). (**E**) Still images of RPE1 cells transfected with NES-A2E (top) or A2E-ARHGAP29 (bottom) imaged live by spinning disk confocal microscopy. Scale bars 10 µm. (**F–H**) Migration tracks (**F**), migration speed (**G**), and mean squared displacement (**H**) of RPE1 cells transfected with A2E-ARHGAP29 or NES-A2E. Quantification was performed on three independent experiments and a total of ~60 cells per sample. Statistical significance in (**G**) was calculated using an unpaired *t*-test; ***p≤0.001. Figure 6—source data 1.Original western blots shown in Figure 6A used for the quantification of data shown in Figure 6B. Figure 6—source data 2.Original pMLC western blots shown in Figure 6A used for the quantification of pMLC levels shown in Figure 6B.

A number of proteins identified in the Cav1 interactome had previously not been linked to caveolae (Figure 4E and H). One of these proteins, the RhoGAP ARHGAP29, has been shown to regulate mechanotransduction in a YAP-dependent manner and to control cancer cell migration and invasion by suppressing actin polymerisation through a Rho/LIMK/cofilin pathway (Meng et al., 2018; Qiao et al., 2017). YAP also regulates caveolar gene transcription and caveolae formation (Rausch et al., 2019), and there is evidence that caveolae, in turn, regulate YAP (Moreno-Vicente et al., 2019; Rausch et al., 2019). This might suggest a functional link between caveolae, ARHGAP29-mediated Rho signalling, and the HIPPO/YAP pathway. To test this, we downregulated ARHGAP29 or Cav1 in RPE1 cells using specific siRNAs (Figure 6A and B). Interestingly, ARHGAP29, YAP, and pS127 YAP levels (a common readout for HIPPO pathway activity) were significantly increased in Cav1 siRNA cells. In addition, the levels of phosphorylated myosin light chain (pMLC, a common readout for myosin-II activation) were markedly decreased in Cav1 siRNA cells. By contrast, ARHGAP29 siRNA transfected cells showed a significant increase in Cav1 Y14 phosphorylation, but pMLC levels were not significantly different to control cells. To test whether the increase in ARHGAP29 protein observed in Cav1 siRNA cells was due to elevated YAP-mediated gene transcription, we performed qPCR analyses. YAP and YAP target gene expression [CYR61 (CCN1), CTGF (CCN2), ANKRD1] as well as ARHGAP29 mRNA levels were unchanged in Cav1 knockdown cells (Figure 6—figure supplement 1). Taken together these data suggest a functional link between caveolae and ARHGAP29, which appears to be independent of YAP signalling.

**Figure 6—figure supplement 1.**
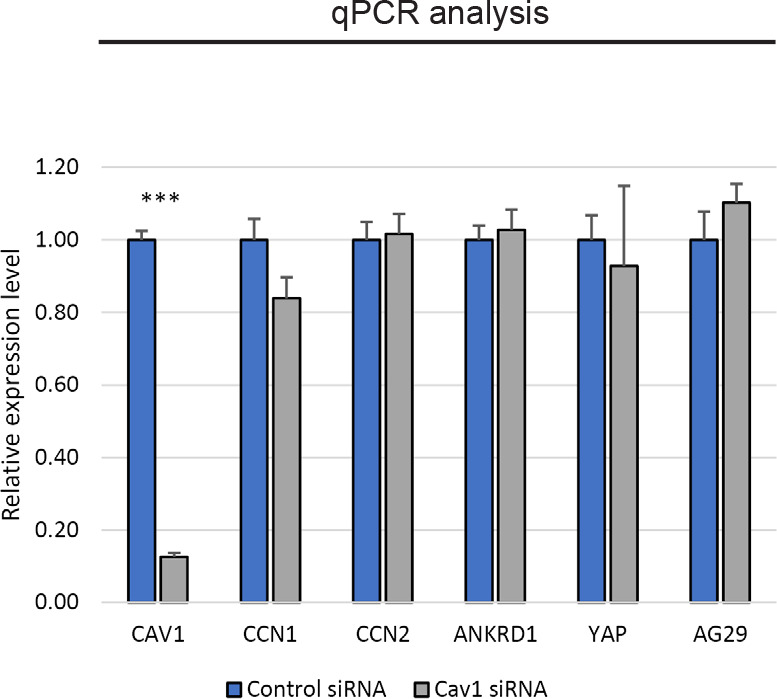
Knockdown of Cav1 in RPE1 cells does not affect the transcription of YAP or YAP target genes. qPCR analysis of control and caveolin-1 siRNA-transfected RPE1 cells. Data is derived from three independent technical replicates. Error bars indicate SEM. ***p≤0.001.

**Figure 6—figure supplement 2.**
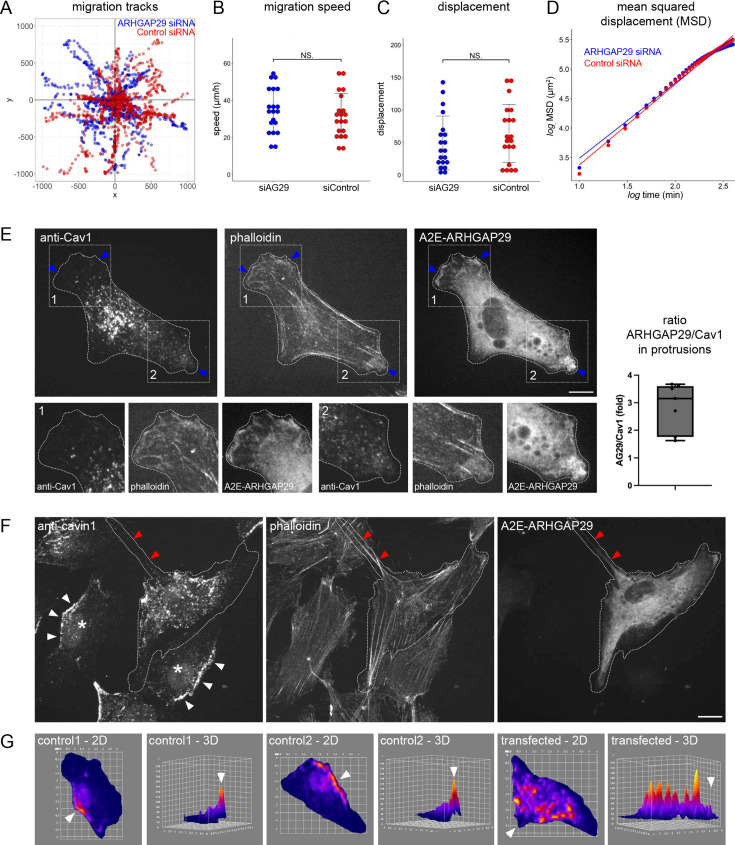
Overexpression and siRNA-mediated knockdown of ARHGAP29. (**A–D**) Migration tracks (**A**), migration speed (**B**), displacement (**C**), and mean squared displacement (**D**) of RPE1 cells transfected with esiRNAs against ARHGAP29 or non-targeting esiRNAs. Quantification was performed on two independent experiments. (**E**) RPE1 cells transfected with A2E-ARHGAP29 were fixed and stained with anti-Cav1 antibodies and fluorescently labelled phalloidin (Alexa Fluor 555). Scale bar 10 µm. Note that cortical areas enriched in ARHGAP29 show reduced Cav1 signals. The fluorescence intensities of ARHGAP29 and Cav1 in such cortical areas were measured and normalised against the intensities on the whole cell level. The ARHGAP29/Cav1 ratio is plotted, showing an approximately threefold enrichment of ARHGAP29 over Cav1. (**F**) RPE1 cells transfected with A2E-ARHGAP29 were fixed and stained with anti-PTRF/cavin1 antibodies and fluorescently labelled phalloidin (Alexa Fluor 555). Asterisks (*) indicate two untransfected control cells. White arrowheads indicate enrichment of PTRF/cavin1 at the cell rear. Red arrowheads indicate actin and ARHGAP29 at the cell rear. Scale bar 10 µm. (**G**) 2D and 3D heat maps of two untransfected control cells (asterisks in **A**) and an A2E-ARHGAP29 transfected cells. White arrowheads indicate the position of the cell rear. Note the loss of PTRF/cavin1 enrichment at the cell rear in cells transfected with A2E-ARHGAP29.

**Figure 6—video 1.** Time-lapse microscopy of RPE1 cells transfected with an NES-APEX2-EGFP plasmid.

**Figure 6—video 2.** Time-lapse microscopy of RPE1 cells transfected with an APEX2-EGFP-ARHGAP29 plasmid.

**Figure 6—video 3.** Time-lapse microscopy of RPE1 cells transfected with an APEX2-EGFP-ARHGAP29 plasmid.

Next we analysed ARHGAP29 siRNA-transfected cells using fixed and live-cell microscopy. The localisation of Cav1 and PTRF/cavin1 was not notably altered in ARHGAP29 siRNA-transfected cells (data not shown). In addition, cells with reduced ARHGAP29 expression showed normal cell migration behaviour compared to control siRNA transfected cells (Figure 6—figure supplement 2A–D). The lack of a migration phenotype may be explained by residual ARHGAP29 activity (i.e., incomplete knockdown) or functional redundancy (i.e., compensation by other related RhoGAPs). We therefore analysed the subcellular distribution of Cav1 and PTRF/cavin1 in cells overexpressing moderate levels of ARHGAP29. Only cells with an intact actin cytoskeleton and normal cell shape were considered for the analysis. Transfected A2E-ARHGAP29 (Figure 6—figure supplement 2E and F) was localised primarily in the cell cytoplasm, with occasional enrichment in lamellipodia-like protrusions. Interestingly, protrusions enriched in ARHGAP29 exhibited a clear reduction in Cav1 staining intensity. When normalised against total cellular protein levels, such protrusions showed an approximately threefold enrichment of ARHGAP29 over Cav1 (Figure 6—figure supplement 2E). In addition, in cells overexpressing ARHGAP29 Cav1 was concentrated in the cell centre rather than being polarised to the rear as observed in cells transfected with the NES-A2E control plasmid (Figure 6C). Quantification of this phenotype revealed that overexpression of A2E-ARHGAP29 indeed perturbed the polarised rear distribution of Cav1 (Figure 6D). The localisation of PTRF/cavin1 was similarly affected (Figure 6—figure supplement 2F and G). These data suggest that ARHGAP29 regulates the polarised distribution of caveolae to the cell rear.

We then investigated the effect of ARHGAP29 overexpression on RPE1 cell migration using time-lapse microscopy (Figure 6E). Control-transfected RPE1 cells showed stable front-rear polarity and migrated randomly but persistently, similar to non-transfected cells (Figure 6E and F, Figure 6—video 1). By contrast, cells overexpressing A2E-ARHGAP29 established multiple random protrusions and failed to specify a stable front-rear axis, resulting either in a complete loss of cell motility (Figure 6E and F, Figure 6—video 2) or in short migration tracks and frequent reversals in direction (Figure 6—video 3). Consequently, ARHGAP29-overexpressing cells showed markedly reduced migration velocities and mean squared displacements compared to control-transfected cells (Figure 6G and H). Taken together the data indicate that ARHGAP29 negatively regulates caveolae rear localisation and RPE1 cell migration.

## Discussion

### Spatially restricted proteomics reveals a membrane-tension-sensitive Cav1 interactome

Using APEX2-mediated proximity proteomics and PLAs, we have identified a distinct Cav1 interactome that is highly sensitive to membrane tension. The data suggest that caveolae link changes in membrane tension to the control of the rear-localised actin cortex, integrin/focal adhesion turnover, and Rho/ROCK signalling (see below). Interestingly, several known mechanotransducers and cell migration regulators including filamins, cortactin, and utrophin were associated with Cav1, suggesting that caveolae integrate mechanosignalling at the rear of migrating cells. We note that the Cav1 interactome is enriched in proteins that are present at but not necessarily exclusive to the cell rear. Given the abundance of caveolae at the rear, the majority of the proteins identified in the interactome are likely to be associated with the rear-localised pool of caveolae. However, Cav1-APEX2 molecules localised elsewhere in the cell, for example in the leading edge, the Golgi apparatus, or on transport vesicles do contribute to the proteome. Therefore, the Cav1 interactome is not a specific interactome of caveolae at the cell rear but rather represents a composite cell-wide interactome of Cav1 oligomers and fully assembled caveolae.

Our proteomics and PLA data show that cavins, EHD2, as well as Pacsin2 are released from Cav1 scaffolds in cells exposed to a brief hypotonic shock. This underscores existing models suggesting that the caveolar coat is rapidly disassembled at high membrane tension (Del Pozo et al., 2021). The data also revealed that the entire caveolar coat structure is re-established within 30 min of recovery from the shock, demonstrating that caveolae assembly and disassembly are remarkably dynamic. It is worth noting that flat Cav1 scaffolds have recently been shown to contain detectable levels of cavins and that EHD2 and Pacsin2 are associated both with flat and curved caveolae (Matthaeus et al., 2022). It is possible, therefore, that the tension-induced conformational changes in the caveolar coat are more subtle than current models suggest. Nonetheless, our work clearly shows that membrane tension has a profound effect on caveolae and that such changes are quantifiable. Altogether, we suggest that membrane tension either dissociates the caveolar coat partially or induces a substantial structural remodelling of many but not all caveolae. One notable caveat of our approach is that the level of tension induced by hypo-osmotic shock likely exceeds that exerted at the rear of the cell. Hence, it will be important to address in future how much membrane tension actually changes at the cell rear as cells migrate, and whether these changes are sufficient to cause caveolae disassembly. We also obtained a list of proteins that became selectively enriched with Cav1 at high membrane tension (Figure 4—figure supplement 1). This proximity proteome is composed of proteins with diverse cellular functions ranging from protein translation (ribosomal proteins) to membrane trafficking. Whether any of these proteins are involved in mediating a Cav1-dependent mechanosignalling response, for instance, via the recently proposed caveolae-independent Cav1 scaffolds termed dolines (Lolo et al., 2023), remains to be tested.

### Membrane tension and actomyosin contractility regulate caveolae rear localisation

We show using fixed and live-cell imaging that caveolar proteins are enriched at and stably associated with the rear of migrating RPE1 cells. Although we cannot discriminate between flat and invaginated caveolae at the resolution provided by diffraction-limited microscopy, large clusters of morphologically distinct caveolae and interconnected caveolar networks were observed at the RPE1 cell rear using electron tomography. Such a polarised distribution of caveolae has been demonstrated in many other migratory cell types (Grande-García and del Pozo, 2008; Grande-García et al., 2007; Hetmanski et al., 2019; Joshi et al., 2008). What is the mechanism driving caveolae formation specifically at the cell rear? Several studies have shown that low membrane tension favours caveolae formation (Del Pozo et al., 2021; Golani et al., 2019; Sinha et al., 2011). This suggests that the formation of caveolae specifically at the rear of migrating cells is the result of a relatively stable front-rear asymmetry in membrane tension. Such membrane tension gradients, with low tension at the rear and higher tension at the front, have indeed been observed in fast-moving fish keratocytes (Lieber et al., 2015; Lieber et al., 2013). However, whether stable membrane tension gradients are a general feature of migrating cells is not entirely clear, and whether membrane tension rapidly equilibrates across the cell (e.g. from the front to the rear) or can be locally confined has been the subject of much debate (De Belly et al., 2023; Shi et al., 2018; Sitarska and Diz-Muñoz, 2020). Using fluorescence lifetime imaging with the Flipper-TR membrane tension probe, the Roux lab recently reported that stable front-rear membrane tension gradients indeed exist in many migrating cells, including RPE1 cells (García-Arcos et al., 2024). Tension gradients were even observed in non-migrating cells and were dependent upon a dynamic F-actin network, cell–substrate adhesions, and membrane–cortex attachments. The study provides strong support that membrane tension gradients are a common feature of adherent cells and that the migration mode determines how such gradients are established.

Hetmanski et al. reported that A2780 cells exhibit a front/rear membrane tension gradient only when cells migrate in an ECM rigidity gradient (Hetmanski et al., 2019). Both inhibition of Rho signalling by Y-27632 and expression of a constitutively active ezrin mutant (which enhances membrane–cortex interactions at the cell rear) increased rear membrane tension, prevented the accumulation of cavin1 and Cav1 at the rear, and blocked cell rear retraction. The role of contractility in concentrating caveolae at the rear is also evident in our time-lapse movies, which suggest that caveolae (cavins) become concentrated at the rear when cells retract their trailing edge. Such sudden rear retractions are likely the result of bursts of contractility that pull on the rear membrane, lowering tension at the rear and promoting caveolae formation. In addition, cavins were frequently aligned in ‘filamentous stripes’ when cells establish front/rear polarity or reorient their front/rear axis, indicating a physical linkage between caveolae and F-actin fibres. Caveolae are indeed attached to and aligned along actin filaments (Echarri and Del Pozo, 2015; Richter et al., 2008; Stoeber et al., 2012) and depolymerisation of the actin cytoskeleton or inhibition of actomyosin contractility affects caveolae distribution and dynamics (Hetmanski et al., 2019; Shi et al., 2021) and blocks caveolae rear localisation (Hetmanski et al., 2019). Altogether our data and a large body of existing literature indicate that caveolae are associated with the F-actin cytoskeleton and that actomyosin-driven contraction and a front/rear membrane tension gradient promote caveolae formation specifically at the rear.

### A model of caveolae function and dynamics at the cell rear

**Figure 7.**
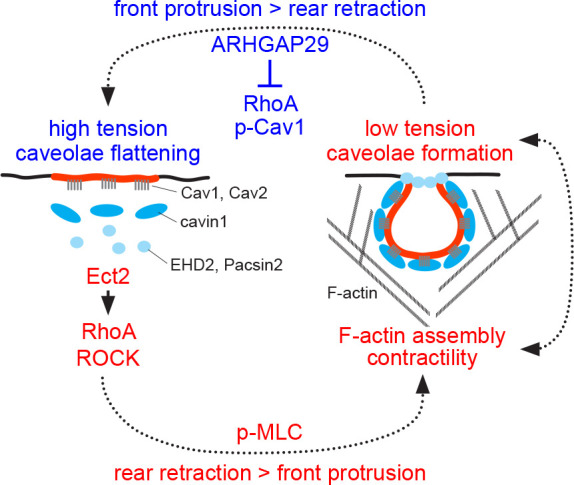
Model of caveolae dynamics and function at the rear of migrating cells. Low membrane tension promotes caveolae formation at the cell rear, whilst high membrane tension causes caveolae to flatten out, which is accompanied by the dissociation of cavin1/PTRF, EHD2, and Pacsin2 from membrane-embedded Cav1 scaffolds. The linkage between the cortical F-actin network and Cav1 scaffolds is also lost at high membrane tension. Caveolae or Cav1 scaffolds promote RhoA/ROCK signalling, MLC phosphorylation, and cell rear retraction, possibly by recruiting the RhoGEF Ect2 and ROCK to the cell rear. ARHGAP29 may be recruited to the cell rear at low membrane tension to suppress RhoA signalling, leading to reduced Cav1 Y14 phosphorylation, increased membrane tension, and caveolae flattening.

A putative model of how membrane tension and caveolae regulate actomyosin contractility and cell rear retraction is shown in Figure 7. The model assumes that a stable membrane tension gradient exists in RPE1 cells (García-Arcos et al., 2024), promoting caveolae formation at the rear. Considering evidence that protrusion at the front is coupled to retraction at the rear and that membrane tension can propagate across the cell (De Belly et al., 2023; Houk et al., 2012; Mueller et al., 2017; Sitarska and Diz-Muñoz, 2020; Tsai et al., 2019), it can be inferred that at short time scales absolute membrane tension values fluctuate at both the rear and the front. This would suggest that caveolae assembly and disassembly at the rear is coordinated with or driven by the protrusion/retraction cycle: When rear tension drops (e.g. in response to a sudden rear retraction), the formation of caveolae and caveolar networks is favoured. When tension at the rear builds up (e.g. when front protrusion is dominant over rear retraction), caveolae flatten out. Since caveolae and caveolar networks substantially increase the effective membrane surface (Golani et al., 2019) and also provide a unique topology and curvature, their dynamic and reversible nature might not only be critical for buffering membrane tension, but also play an important role in regulating the recruitment of signalling proteins to the rear.

Previous studies have demonstrated a critical role for Rho/ROCK signalling in caveolae formation and cell rear retraction (Hetmanski et al., 2019; Hetmanski et al., 2021). Knockdown of RhoA, ROCK1, or the rear-localised RhoGEF Ect2 in A2780 cells blocked caveolae rear localisation and impeded cell migration in durotactic gradients. Caveolae, in turn, were required for the recruitment of Ect2 to the rear, for RhoA activation, and for cell rear retraction, suggesting that caveolae and the actomyosin system control cell rear retraction via a positive feedback loop (Hetmanski et al., 2019). Our data suggest that ROCK1 is recruited to Cav1 at high membrane tension (i.e. under conditions that promote caveolae flattening) and that ROCK activity is required for caveolae reassembly after hypo-osmotic shock. In addition, we show that the loss of Cav1 leads to a drastic reduction in MLC phosphorylation, indicating that Cav1 is critical for myosin-II activation. Altogether this suggests that Cav1 scaffolds recruit Ect2 and ROCK1 to the cell rear to promote caveolae formation and actomyosin contractility. As discussed below, the function of ARHGAP29 might be to interrupt this feedback loop by suppressing RhoA signalling at the rear.

### A putative functional role of ARHGAP29 at the cell rear

We provide evidence that ARHGAP29 is associated with Cav1 in a membrane tension-dependent manner and that overexpression of ARHGAP29 suppresses caveolae rear localisation and RPE1 cell migration. Since caveolae are highly enriched at the cell rear, we expected that ARHGAP29 would (at least partially) be associated with the rear. However, both in fixed and live RPE1 cells ARHGAP29 appeared mostly cytoplasmic, with no obvious rear bias. This was somewhat unexpected as ARHGAP29 is localised to the plasma membrane in several cell types (Post et al., 2015; Rogg et al., 2023; Tan et al., 2020b; Tan and Zaidel-Bar, 2015). Closer inspection revealed that a fraction of ARHGAP29 was present in lamellipodia-like protrusions, which interestingly were largely devoid of Cav1. This inverse spatial correlation suggests that ARHGAP29 can locally suppress caveolae assembly or prevent caveolae recruitment into such areas. This effect is likely due to the local inhibition of RhoA and can explain the caveolae rear polarity and cell migration defects we observe in cells overexpressing ARHGAP29. The apparent absence of ARHGAP29 from the cell rear at steady state then prompts the question why ARHGAP29 was identified in the Cav1 proteome with such specificity and reproducibility in the first place. This can be explained by the way the APEX2 enzyme works. Proximity biotinylation with APEX2 is extremely sensitive and is restricted to a labelling radius of ~20 nm (Hung et al., 2016). The labelling reaction is conducted on live and intact cells at room temperature (RT) for 1 min. Although 1 min appears short, dynamic cellular processes occur at the time scale of seconds and are ongoing during the labelling reaction. It is conceivable, therefore, that within this 1 min time frame, ARHGAP29 cycles on and off the rear membrane (kiss and run). This might allow ARHGAP29 to be biotinylated by Cav1-APEX2, resulting in its identification by MS. Interestingly, ARHGAP29 contains an N-terminal F-BAR domain that is required for membrane targeting and that can sense changes in membrane curvature. Work in *Caenorhabditis elegans* suggests that membrane recruitment of SPV-1, the worm orthologue of ARHGAP29, is favoured at low membrane tension, leading to RhoA inactivation at the cortex. Increased membrane tension releases ARHGAP29 from the membrane, permitting cortical RhoA activation and contractility (Tan and Zaidel-Bar, 2015). This is line with our proteomics data and suggests that subtle changes in membrane tension might control ARHGAP29 levels at the cell rear. Recruitment of ARHGAP29 to the rear at low membrane tension (which presumably occurs immediately following the retraction of the rear) would transiently suppress Rho-mediated contractility, providing a potential mechanism to terminate rear retraction and to permit a new protrusion/retraction cycle.

Although our data do suggest a reciprocal functional connection between Cav1 and ARHGAP29, how and where ARHGAP29 functions in RPE1 cells remains unclear. Hence, the model proposed above is largely speculative and alternative scenarios are conceivable. Firstly, the proposed model suffers from the lack of strong evidence that ARHGAP29 is actually recruited to and active at the rear. Instead of transiently operating at the rear where RhoA-GTP levels are relatively high, it is equally possible that ARHGAP29 inhibits Rho activation at the cell front, similar to what has been suggested for podocytes (Rogg et al., 2023). In this scenario, ARHGAP29 might be involved in establishing a Rac/Rho activity gradient along the front/rear axis (Ridley et al., 2003) rather than dampening RhoA activity at the rear. Secondly, we find that siRNA-mediated silencing of Cav1 expression results in a drastic reduction in cellular pMLC levels. By contrast, and unexpectedly, downregulation of ARHGAP29 had little or no effect on pMLC levels. This suggests that Cav1 and ARHGAP29 control actin dynamics in fundamentally different ways. Whilst Cav1 promotes cell rear retraction via RhoA/ROCK/MLC signalling (Hetmanski et al., 2019; Shi et al., 2021), ARHGAP29 may control the stability or turnover of F-actin networks by suppressing a RhoA/LIMK/cofilin pathway (Qiao et al., 2017). Cofilin-driven actin depolymerisation plays an important role in cell motility (Bravo-Cordero et al., 2013), although there is conflicting data over whether cofilin is active at the leading edge (Bisaria et al., 2020) or at the cell rear (Mseka and Cramer, 2011). Since myosin-II and cofilin crosstalk to control actomyosin dynamics (Wiggan et al., 2012), the functions of ARHGAP29 and caveolae may be intertwined in a complex manner. Thirdly, we find that downregulation of ARHGAP29 significantly increases Cav1 Y14 phosphorylation, whilst downregulation of Cav1 stabilises or promotes the expression of ARHGAP29. Previous studies have shown that Y14 phosphorylation is dependent upon ROCK and Src activity and a role in the control of focal adhesion dynamics, cell migration, and caveolae-mediated mechanosignalling has been proposed (Joshi et al., 2012; Joshi et al., 2008; Meng et al., 2017; Shi et al., 2021; Zimnicka et al., 2016). Taken together this suggests that ARHGAP29 interferes with RhoA/ROCK or Src signalling to suppress Cav1 phosphorylation. Caveolae, in turn, somehow dampen ARHGAP29 expression to reinforce a positive feedback loop that promotes actomyosin-mediated cell rear retraction.

In conclusion, our data and previous work (Hetmanski et al., 2019; Hetmanski et al., 2021) suggest that caveolae act as a rear-localised membrane tension sensor that links the F-actin cortex and actomyosin contractility to cell rear retraction. The caveolar interactome identified here will be instrumental in dissecting the molecular machinery and mechanisms underlying caveolae-mediated mechanotransduction in migrating cells.

## Materials and methods

**Key resources table:** 

Reagent type (species) or resource	Designation	Source or reference	Identifiers	Additional information
Cell line (human)	hTERT RPE-1	ATCC	ATCC CRL-4000 RRID:CVCL_4388	Human retinal pigment epithelium
Cell line (human)	hTERT RPE-1 Cavin1-APEX2-EGFP	Ludwig, 2020	NA	Stable transfection
Cell line (human)	hTERT RPE-1 Cavin3-miniSOG-mCherry	Ludwig et al., 2013	NA	Stable transfection
Cell line (human)	hTERT RPE-1 NES-A2E	This study	NA	Stable transfection
Cell line (human)	hTERT RPE-1 Cav1-A2E	This study	NA	Stable transfection
Transfected construct (human)	NES-A2E	Tan et al., 2020b	NA	Recombinant DNA
Transfected construct (human)	Cav1-A2E	Ludwig et al., 2016	NA	Recombinant DNA
Chemical compound, drug	DMEM:F12 1:1 Mixture	Westburg	Cat# LO BE04-687F/U1	NA
Chemical compound, drug	Geneticin Selective Antibiotic (G418 Sulfate)	Fisher Scientific	Cat# 10131027	500 µM
Peptide, recombinant protein	Gibco Fibronectin Bovine Protein, Plasma	Fisher Scientific	Cat# 33010018	1:200
Other	Lipofectamine 3000 Reagent	Thermo Fisher Scientific	Cat# L3000015	transfection reagent
Other	Dharmafect 1	Thermo Fisher Scientific	Cat# T-2001	transfection reagent
Peptide, recombinant protein	Streptavidin Alexa Fluor 568	Thermo Fisher Scientific	Cat# S11226 RRID:AB_2315774	IF: 1:1000
Peptide, recombinant protein	Streptavidin-HRP	Thermo Fisher Scientific	Cat# 434323 RRID:AB_2619743	WB: 1:10,000
Commercial assay or kit	Duolink In Situ Orange Starter Kit Mouse/Rabbit	Sigma-Aldrich	Cat# DUO92102-1KT	NA
Chemical compound, drug	Biotin phenol	Iris Biotech	Cat# LS-3500	0.5 mM
Chemical compound, drug	Trolox	Sigma-Aldrich	Cat# 238813	5 mM
Chemical compound, drug	Glutaraldehyde (32%)	Electron Microscopy Sciences (EMS)	Cat# 16220	2%
Chemical compound, drug	Paraformaldehyde (32%)	Electron Microscopy Sciences (EMS)	Cat# 100504–858	1–4%
Chemical compound, drug	Diaminobenzidine (DAB) (Free-Base)	Sigma-Aldrich	Cat# D8001	0.5 mg/ml
Chemical compound, drug	Durcupan ACM resin	Electron Microscopy Sciences (EMS)	Cat# 44610	NA
Chemical compound, drug	Osmium tetroxide (2%)	Electron Microscopy Sciences (EMS)	Cat# 19152	1–2%
Chemical compound, drug	Potassium ferricyanide	Sigma-Aldrich	Cat# 702587	1–2% (w/v)
Peptide, recombinant protein	Sequencing Grade Modified Trypsin	Promega Corporation	Cat# V5111	
Peptide, recombinant protein	Lysyl Endopeptidase, MS Grade	FUJIFILM Wako Pure Chemical Corporation	Cat# 125–05061	
Peptide, recombinant protein	Complete, EDTA-free Protease Inhibitor Cocktail	Roche	Cat# 11873580001	
Peptide, recombinant protein	Thermo Scientific Pierce Streptavidin Magnetic Beads	Fisher Scientific	Cat# 10615204	
Commercial assay or kit	EZQ Protein Quantitation Kit	Fisher Scientific	Cat# R33201	
Antibody	Rabbit monoclonal anti-PARG1 (ARHGAP29)	Invitrogen	Cat# PA5-55336 RRID:AB_2645210	WB: 1:1000
Antibody	Rabbit polyclonal anti-Caveolin-1 (CAV1)	BD Bioscience/ Transduction labs	Cat# 610060 RRID:AB_397472	WB: 1:10,000 IF/PLA: 1:500
Antibody	Mouse monoclonal anti-Caveolin-1 pY14	BD Bioscience/ Transduction labs	Cat# 611339 RRID:AB_398863	WB: 1:1000
Antibody	Rabbit polyclonal anti-Cavin-1 (PTRF)	Abcam	Cat# ab48824 RRID:AB_882224	WB: 1:5000 IF/PLA: 1:500
Antibody	Goat polyclonal anti-EHD2	Abcam	Cat# ab23935 RRID:AB_2097328	WB: 1:2000
Antibody	Mouse monoclonal anti-Filamin 1 (E-3) (FLNA)	Santa Cruz Biotechnology	Cat# sc-17749 RRID:AB_627606	IF/PLA: 1:100
Antibody	Mouse monoclonal anti-Cortactin (clone 4F11)	Merck Millipore	Cat# 05–180 RRID:AB_309647	IF/PLA: 1:100
Antibody	Mouse monoclonal anti-Rock-1 (G-6)	Santa Cruz Biotechnology	Cat# sc-17794 RRID:AB_628223	IF/PLA: 1:100
Antibody	Mouse monoclonal anti-HSP27 (F-4) (HSPB1)	Santa Cruz Biotechnology	Cat# sc-13132 RRID:AB_627755	IF/PLA: 1:100
Antibody	Mouse monoclonal anti-GFP	Roche	Cat# 11814460001 RRID:AB_390913	WB: 1:2000
Antibody	Rabbit polyclonal anti-GFP	Abcam	Cat# ab290 RRID:AB_2313768	IF/PLA: 1:100
Antibody	Mouse monoclonal anti-YAP	Cell Signalling Technology	Cat# 14074 RRID:AB_2650491	WB: 1:1000
Antibody	Mouse monoclonal anti-YAP pS127	Cell Signalling Technology	Cat# 13008 RRID:AB_2650553	WB: 1:5000
Antibody	Rabbit polyclonal anti-ppMLC2 (Thr18/Ser19)	Cell Signalling Technology	Cat# 3674 RRID:AB_2147464	WB: 1:1000
Antibody	Mouse anti-GAPDH	Santa Cruz Biotechnology	Cat# 47724 RRID:AB_627678	WB: 1:5000
Antibody	Donkey anti-Mouse IgG (H+L) Alexa Fluor 488	Invitrogen	Cat# A-21202 RRID:AB_141607	IF: 1:500
Antibody	Donkey anti-Rabbit IgG (H+L) Alexa Fluor 488	Invitrogen	Cat# A-21206 RRID:AB_2535792	IF: 1:500
Antibody	Donkey anti-Mouse IgG (H+L) Alexa Fluor 555	Invitrogen	Cat# A-31570 RRID:AB_2536180	IF: 1:500
Antibody	Donkey anti-Rabbit IgG (H+L) Alexa Fluor 555	Invitrogen	Cat# A-31572 RRID:AB_162543	IF: 1:500
Antibody	Goat anti-Rabbit IgG (H+L) Alexa Fluor 633	Invitrogen	Cat# A-21071 RRID:AB_2535732	IF: 1:500
Antibody	Goat anti-Mouse IgG (H+L) Alexa Fluor 633	Invitrogen	Cat# A-21052 RRID:AB_2535719	IF: 1:500
Antibody	Goat anti-rabbit IgG (H+L) HRP conjugate	Invitrogen	Cat# A16104 RRID:AB_2534776	WB: 1:5000
Antibody	Goat anti-mouse IgG (H+L) HRP conjugate	Invitrogen	Cat# A16072 RRID:AB_2534745	WB: 1:5000
Recombinant DNA reagent	Caveolin-1 siRNA ON-TARGET Plus SMART pool	Dharmacon	Cat# L-003467-00-0020	100 nM
Recombinant DNA reagent	ARHGAP29 Mission esiRNA	Sigma	Cat# EHU13457	100 nM
Recombinant DNA reagent	FLUC Control Mission esiRNA	Sigma	Cat# EHUFLUC	100 nM
Recombinant DNA reagent	EHD2 For (HindIII)	Integrated DNA Technologies	CGCAAAGCTTCTATGTTCAGCTGGCTGAAGCGG	Forward primer for cloning of EHD2-A2E
Recombinant DNA reagent	EHD2 Rev (EcoRI)	Integrated DNA Technologies	ATACGAATTCTCTCGGCGGAGCCCTTGTGGCG	Reverse primer for cloning of EHD2-A2E
Recombinant DNA reagent	CCN1 (NM_001554.5)	Integrated DNA Technologies	For: TGAAGCGGCTCCCTGTTTTT Rev: TGAGCACTGGGACCATGAAG	primers for qPCR
Recombinant DNA reagent	CCN2 (NM_001901.4)	Integrated DNA Technologies	For: CACCCGGGTTACCAATGACA Rev: GGATGCACTTTTTGCCCTTCTTA	primers for qPCR
Recombinant DNA reagent	ANKRD1 (NM_014391.3)	Integrated DNA Technologies	For: TAGCGCCCGAGATAAGTTGC Rev: GTCTGCCTCACAGGCGATAA	primers for qPCR
Recombinant DNA reagent	YAP (NM_001130145.3)	Integrated DNA Technologies	For: ACTCGGCTTCAGGTCCTCTT Rev: GGTTCATGGCAAAACGAGGG	primers for qPCR
Recombinant DNA reagent	ARHGAP29 (NM_001328664.2)	Integrated DNA Technologies	For: ACATCTAAAGCGGGTAGTAG Rev: AAGGGAGGAGATGGTGATAG	primers for qPCR
Recombinant DNA reagent	CAV1 (NM_001753.5)	Integrated DNA Technologies	For: ACGTAGACTCGGAGGGACA Rev: TCGTACACTTGCTTCTCGCT	primers for qPCR
Recombinant DNA reagent	GAPDH (NM_002046.7)	Integrated DNA Technologies	For: TCGGAGTCAACGGATTTGGT Rev: TGAAGGGGTCATTGATGGCA	primers for qPCR
Software, algorithm	ImageJ/FIJI	NA	https://Imagej.nih.gov/ij RRID:SCR_002285	
Software, algorithm	MaxQuant (version 1.6.7.0)	NA	https://www.maxquant.org/ RRID:SCR_014485	
Software, algorithm	UniProt	NA	https://www.uniprot.org/ RRID:SCR_002380	
Software, algorithm	ProTIGY (version 1.1.7)	Broad Institute, Proteomics Platform	https://github.com/broadinstitute/protigy	
Software, algorithm	Cytoscape	NA	https://cytoscape.org RRID:SCR_003032	
Software, algorithm	BioGRID	NA	https://thebiogrid.org RRID:SCR_007393	
Software, algorithm	STRING	NA	https://string-db.org RRID:SCR_005223	
Software, algorithm	Inkscape	NA	https://inkscape.org/ RRID:SCR_014479	
Software, algorithm	R (version 4.0.3)	NA	https://www.r-project.org/ RRID:SCR_001905	

### DNA constructs and bacterial strains

The cavin3-miniSOG-mCherry, Cav1-APEX2-EGFP, and NES-APEX2-EGFP vectors were described previously (Ludwig et al., 2013; Ludwig et al., 2016; Tan et al., 2020b). To produce EHD2-A2E, human EHD2 cDNA was cloned into the N1-A2E vector (Tan et al., 2020b) using PCR and *HindIII* and *XhoI* sites (see Key Resources Table for oligonucleotides used). To produce EGFP-APEX2-ARHGAP29, the ARHGAP29 cDNA was sub-cloned into the C1-A2E vector by PCR using HA-ARHGAP29 (Addgene plasmid #104154) as a template (Tan et al., 2020b). NEB10-beta competent *Escherichia coli* (NEB, Cat# C3019I) were transformed for plasmid amplification and then cultured in LB Agar. Single colonies were isolated and further expanded in LB broth before plasmid purification with miniprep (QIAGEN) following the manufacturer’s protocol. All plasmids were verified by Sanger sequencing.

### Antibodies

All antibodies used are listed in the Key Resources Table.

### RPE1 cell culture and transfection

Human (h)TERT-RPE1 (CRL-4000) cells were purchased from ATCC and tested for mycoplasma on a regular basis using a PCR-based mycoplasma test kit. RPE1 cells were cultured in DMEM-F12 supplemented with 10% FBS, 100 units/ml penicillin, and 100 μg/ml streptomycin (Gibco) at 37°C and 5% CO_2_. Transfections were performed in 6-well plates with the cells in a sub-confluent condition. Cells were transfected with Lipofectamine 3000 (Thermo Fisher Scientific) using 1 µg plasmid DNA per well. Stable cell lines were generated by selection in complete medium containing 400 µg/ml geneticin (Gibco) for at least 14 days. Stable cell lines were maintained in media containing 200 µg/ml geneticin and occasionally subjected to fluorescence-activated cell sorting (FACS) to maintain populations in which ~40% of cells expressed Cav1-A2E or NES-A2E in the respective lines.

### Hypo-osmotic shock and inhibitors

For hypo-osmotic shock, cells were shifted from normal growth medium to hypo-osmotic medium (10% DMEM/90% deionised water) for 5 min and then immediately collected for the hypo-osmotic condition, or switched back to normal medium for 30 min to let cells recover from the osmotic shock. For the ROCK inhibition assays, the 5 min shock was performed in the presence or absence of 10 µM Y27632 (DMSO (1 µl/ml) was used as a control), followed by 30 min recovery in the presence or absence of the inhibitor.

### Immunofluorescence

RPE1 cells grown on fibronectin-coated glass coverslips were washed twice in PBS and fixed in 4% paraformaldehyde for 15 min. After further washing, cells were permeabilised with 0.1% Triton X-100 in PBS for 5 min. Blocking was performed with 10% FBS in PBS for 1 hr at RT. Samples were incubated for 1 hr at RT with primary antibodies diluted in 0.1% bovine serum albumin (BSA), 0.01% Tween-20 in PBS (antibody buffer). After three washes with antibody buffer, samples were further incubated with the appropriate fluorescent-conjugated secondary antibody for 1 hr at RT. After three last washes in PBS, coverslips were mounted in Vectashield Antifade Mounting Medium containing DAPI (Vector Laboratories). Slides were imaged either on a spinning disk microscope (CorrSight, Thermo Fisher Scientific) equipped with an Orca R2 CCD camera (Hamamatsu) or on a laser scanning confocal microscope (LSM880 FastAiry, Carl Zeiss). Images were acquired with a ×63 oil immersion Plan-Apochromat objective (NA = 1.4, Zeiss) and standard filter sets. Confocal stacks were processed in ImageJ/FIJI.

### Electron microscopy

For electron microscopy, cells were fixed in 2.5% glutaraldehyde (EM grade, EMS) in 0.1 M cacodylate buffer pH 7.4 (CB) for 1 hr. After several washes in CB, cells were incubated in 0.5 mg/ml diaminobenzidine free-base (DAB, Sigma-Aldrich) and 0.5 mM H_2_O_2_ in CB for 5–10 min, post-fixed in 1% osmium tetroxide (EMS), 1% (v/w) potassium ferricyanide (Sigma-Aldrich) in CB, dehydrated using a graded ethanol series, and further processed for TEM as described previously (Ludwig, 2020). For CLEM, areas containing the cells of interest were sawed out using a jeweller’s saw and sectioned parallel to the substratum using a diamond knife. 70–80 nm semithin serial sections were picked up on formvar- and carbon-coated EM slot grids and imaged on a TecnaiT12 TEM (Thermo Fisher Scientific) operated at 120 kV equipped with a 4k × 4k Eagle camera (Thermo Fisher Scientific). MiniSOG labelling and 3D tomography were described previously (Ludwig et al., 2013). Tomograms were reconstructed using weighted back projection and further analysed in IMOD/etomo.

### Proximity ligation assay

In situ PLA was performed with DuoLink PLA kit (Sigma-Aldrich) following the manufacturer’s protocol. Briefly, after fixation and permeabilisation steps described above, cells were first incubated in Duolink Blocking Solution for 1 hr at 37°C and then with the appropriate mix of primary antibodies for 1 hr at RT. After washes, coverslips were incubated with PLA probe solution for 1 hr at 37°C. Ligation and amplification steps were performed at 37°C for 30 and 100 min, respectively. Coverslips were mounted with Duolink PLA Mounting Medium with DAPI.

### PLA imaging and quantification

Slides were imaged on a Zeiss LSM 880 confocal microscope and images were acquired with a ×63 oil immersion Plan-Apochromat objective 1.4 numerical aperture (Zeiss) and standard filter sets. Image analysis was performed with ImageJ/FIJI according to published protocols. Briefly, single-stack images were split in separate channels. The green channel was used for cell boundaries identification while the red channel for PLA signal retrieval. Thirty different cells from three independent experiments were used for quantification. The dot plots represent the number of PLA signals per cell.

### Proximity biotinylation

The APEX2 labelling protocol was adapted from previously published papers (Hung et al., 2016; Tan et al., 2020a; Tan et al., 2020b). Briefly, cells were grown on glass coverslips (for IF) or on plastic dishes (for MS and WB). Cells were incubated for 30 min at 37°C in media containing 0.5 mM biotin phenol (Iris Biotech). After two washes in PBS, the labelling reaction was performed for 1 min with 0.5 mM H_2_O_2_ in PBS. Due to the higher expression level of NES-A2E compared to Cav1-A2E, we adjusted the hydrogen peroxide concentration to obtain an overall biotinylation intensity comparable to that obtained in the Cav1-A2E cell line. To quench the reaction, three washes with a quencher solution (5 mM (±)–6-hydroxy-2,5,7,8-tetramethylchromane-2-carboxylic acid (Trolox), 10 mM sodium ascorbate, 10 mM sodium azide in PBS) were performed. Cells were then further processed either for IF or protein extraction.

### Protein extraction and western blotting

Cells were incubated in lysis buffer (1% SDC in 50 mM ammonium bicarbonate [AmBic]) and scraped using a cell scraper. Lysates were then sonicated (three cycles of pulse sonication for 20 s) and clarified (centrifugation at 16,000 × *g* for 30 min at 4°C). Protein concentration was assessed either with Bradford’s assay or with EZQ protein quantification assay (Thermo Fisher Scientific) and then kept at –80°C until further processing. Proteins were separated on precast 4–12% polyacrylamide gels and blotted on polyvinylidene difluoride (PVDF) membranes. Membranes were then dehydrated in methanol for 10 s and allowed to dry for 1 hr. Membranes were incubated with primary antibodies diluted in 2% BSA in PBST (PBS, 0.2% Tween-20). After 3 × 10 min washes in PBST, membranes were incubated with HRP-conjugated secondary antibody diluted in 5% fat-free milk in PBST. After further washes, membranes were developed with chemiluminescent substrate. To test biotinylation efficiency, membranes were incubated with streptavidin-HRP conjugated diluted 1:10,000 in PBST containing 2% BSA for 1 hr at RT and then washed five times with PBST before development.

### Streptavidin purification and on-bead digestion

Streptavidin Beads (Pierce Streptavidin magnetic beads, Thermo Fisher Scientific) were washed twice with cold lysis buffer before incubation with protein lysates (50 µl bead slurry per 1 mg lysate) for 1.5 hr at 4°C on a rotating wheel. Beads were then washed twice with cold lysis buffer, and then once with each of the following washing buffers: 1 M KCl, 0.1 M of Na_2_CO_3_, 2 M urea in 50 mM AmBic pH 8. The last two washes were performed with cold 50 mM AmBic pH 8. Beads were then re-suspended in digestion buffer (2 M urea in 50 mM AmBic). Protein reduction was performed by adding 1 M DTT to reach 10 mM final concentration, and incubating for 45 min at 37°C and 800 rpm. After incubation with Iodocaetamide (25 mM final concentration, 30 min at RT and 800 rpm), alkylation reaction was stopped by incubating the samples at natural light for 2 min. Protein digestion was performed in a two-step reaction: samples were pre-digested with 0.5 µg LysC for 4 hr at 37°C and 800 rpm and then digested with 1 µg trypsin O/N at 37°C and 800 rpm. Peptides were then acidified (pH 2) by adding formic acid to 1% final concentration and centrifuged for 15 min at 16,000 rpm at 4°C to remove any residues of beads before proceeding with desalting on Sep-Pak µ-C18 Elution Plates. Peptides were then dried on a speed vac for 2 hr and re-suspended in 0.1% formic acid prior to LC-MS.

### Mass spectrometry

LC-MS/MS was performed using an Ultimate 3000 RSLCnano (Thermo Fisher Scientific) LC system equipped with an Acclaim PepMap RSLC column (15 cm × 75 μm, C18, 2 μm, 100 Å, Thermo Fisher Scientific). Peptides were eluted at a flow rate of 300 nl/min in a linear gradient of 2–35% solvent B over 70 min (solvent A: 0.1% formic acid; B: 0.1% formic acid in acetonitrile [ACN]), followed by a 5 min washing step (90% solvent B) and a 10 min equilibration step (2% solvent B). Mass spectrometry was performed on a Q-Exactive Plus mass spectrometer (Thermo Fisher Scientific) equipped with a nano-electrospray source and using uncoated SilicaTips (12 cm, 360 μm o.d., 20 μm i.d., 10 μm tip i.d.) for ionisation, applying a 1500 V liquid junction voltage at 250°C capillary temperature. MS/MS analysis were performed in data-dependent acquisition mode, and the 12 most intense parent ions were fragmented with a resolving power of 17,500 (at 200 m/z). Automatic gain control, maximum fill time, and dynamic exclusion were set to 1e6, 60 ms, and 30 ms, respectively.

### Data analysis for mass spectrometry

Protein quantification was performed with MaxQuant (version 1.6.7.0) using the following parameters: carbamidomethyl cysteine as fixed modification, methionine oxidation and N-acetylation as variable modifications, digestion mode trypsin specific with a maximum of two missed cleavages, and an initial mass tolerance of 4.5 ppm for precursor ions and 0.5 Da for fragment ions. All experiments were performed in triplicates, and the abundance of assembled proteins was determined using label-free quantification (standard MaxQuant parameters) and match between run option was selected. Protein identification was performed searching against the UniProt reference proteome (UniProt ID 9606, downloaded on January 6, 2020). To this database were added two sequences for the detection of the fusion protein: Cav1-CANLF (UniProt id: P33724) and a manually curated fusion protein combining APEX2 (Q43758) and EGFP (P42212). Further processing with Perseus (version 1.6.10.45) was performed according to the following pipeline: removal of common contaminants, reverse and peptides identified only on site-specific modifications, and log2 transformation. Only proteins identified in three out of three replicates of at least one sample were considered for further analysis. Moderated *t*-test was performed with Proteomics Toolset for Integrative Data Analysis (ProTIGY, Broad Institute, Proteomics Platform, https://github.com/broadinstitute/protigy), and a p-value <0.05 was considered statistically significant. Further data analysis and visualisation were performed in R (version 4.0.3). The mass spectrometry data is summarised in Supplementary file 1.

### Generation of the Cav1 interactome

Interactions among the proteins identified in the Cav1 proximity proteome were retrieved from BioGRID version 4.2.193 (April 2020) and the STRING database. For BioGRID entries, only physical protein interactions were considered; methods such as Affinity Capture-RNA, Protein-RNA, and Proximity Label-MS were disregarded. For STRING, the following parameters were used: Active interaction; source: Database; Confidence: >0.4. In addition, direct or indirect protein–protein interactions reported in the primary literature were included (see Supplementary file 2). The protein network was drawn in Cytoscape version 3.7.1.

### Live-cell imaging and cell migration analysis

RPE1 cells were grown on fibronectin-coated glass-bottom dishes (MatTec Corp., Ashland, MA) and imaged live in complete DMEM without Phenol Red on a CorrSight spinning disk microscope (Thermo Fisher Scientific). Imaging was carried out at 37°C, 5% CO_2_, and 90% humidity in a closed atmosphere chamber (IBIDI). Up to five different locations were imaged simultaneously using the multi-stage position function in LA software (Thermo Fisher Scientific). Confocal image stacks (typically 3–4 images with a z-interval of 600–1000 nm) were acquired as 3 × 3 tiles with a ×40 oil objective (NA 1.3, EC Plan Neofluar M27, Zeiss) using the 488 nm laser line (65 mW; iChrome MLE-LFA) and a standard GFP filter set. Typically time-lapse movies were recorded at a frame rate of 5 or 10 min for 6–12 hr (in some cases even longer). Focus was maintained by a hardware autofocus system (Focus Clamp). The laser output power and exposure times were set to a minimum. Tiles were stitched and confocal image stacks converted into maximum intensity projections. Time-lapse recordings were analysed in ImageJ/FIJI (Schindelin et al., 2012; Schneider et al., 2012).

Cell migration data was analysed with the ImageJ Manual Tracking plugin. Twenty-five cells per condition in five individual movies were analysed, and all experiments were performed in triplicate. GFP-positive cells were identified and tracked frame-by-frame by manually clicking on the centre of the nucleus. The X/Y coordinates were then imported into R, and CelltrackR package was used for further analysis (Wortel et al., 2021). Data were normalised to overlay track starting points to zero. The R package was then used to calculate the duration of migration, displacement, track length, speed, and mean squared displacement.

### Quantification of rear localisation

All quantifications were performed in ImageJ/FIJI. For the quantification of Cav1 rear enrichment in fixed cells shown in Figure 1A, the mean pixel intensities at the cell rear and the cell front were measured. The cell rear was outlined manually based on the highest Cav1 signal. An equal size area was then measured at the front of the cell. The rear/front signal intensity ratios for each cell were calculated and plotted.

For the quantification of caveolae rear localisation in time-lapse movies, a 50 pixel wide line was drawn from the rear towards the front along the longest cell axis. The position and vector of the line were adjusted for each frame. Pixel intensities along the line were measured using the Plot Profile function of ImageJ/FIJI and exported into Excel. For the quantification shown in Figure 5D, the pixel intensities along the line were divided into four equal size quadrants - rear, centre rear, centre front, and front. The average pixel intensities in each quadrant were determined and plotted. All pixel intensity values were background subtracted. Graphs were produced in Excel, Prism, or R.

### siRNA transfection

siRNAs specific to human caveolin-1 and ARHGAP29 and non-targeting siRNAs (see Key Resources Table) were transfected at a final concentration of 50–100 nM using Dharmafect (Sigma-Aldrich) according to the manufacturer’s recommendations. Transfected cells were analysed by western blotting 48 hs post-transfection. For immunofluorescence and cell migration analysis, cells were trypsinised 48 hr post-transfection and seeded at low density onto glass coverslips or MatTec dishes, respectively. Cells were imaged 24 hr later.

### qPCR analysis

RNA was extracted using the PureLink RNA Mini Kit (Invitrogen). Genomic DNA was removed using DNAse1 (Thermo Scientific) and cDNA was prepared using Superscript II Reverse Transcriptase (Invitrogen). qPCR was carried out using SsoAdvanced Universal SYBR Green Supermix (Bio-Rad) on a CFX96 Real-Time System (Bio-Rad). Experiments were conducted in technical triplicates. Data was normalised to endogenous GAPDH levels and analysed using the comparative Ct method. Error bars represent the mean ± standard deviation, and a Student’s *t*-test was used to generate p-values (*≤0.05, **≤0.01, ***≤0.001) (qPCR primers are listed in the Key Resources Table).

## Data Availability

The raw mass spectrometry data was deposited in the PRIDE repository under accession PXD026464. The following dataset was generated: MartinE GirardelloR DittmarG LudwigA 2024Spatially restricted proteomics reveals dynamic changes in the caveolin-1 interactome in response to increased membrane tensionPRIDEPXD026464

## Additional files

Supplementary file 1. Mass spectrometry data.

Supplementary file 2. Literature and database entries for the Cav1 interactome analysis.

MDAR checklist

## References

[bib1] Baldassarre M, Razinia Z, Burande CF, Lamsoul I, Lutz PG, Calderwood DA (2009). Filamins regulate cell spreading and initiation of cell migration. PLOS ONE.

[bib2] Bisaria A, Hayer A, Garbett D, Cohen D, Meyer T (2020). Membrane-proximal F-actin restricts local membrane protrusions and directs cell migration. Science.

[bib3] Bravo-Cordero JJ, Magalhaes MAO, Eddy RJ, Hodgson L, Condeelis J (2013). Functions of cofilin in cell locomotion and invasion. Nature Reviews. Molecular Cell Biology.

[bib4] Chai Q, Wang XL, Zeldin DC, Lee HC (2013). Role of caveolae in shear stress-mediated endothelium-dependent dilation in coronary arteries. Cardiovascular Research.

[bib5] Cheng JPX, Mendoza-Topaz C, Howard G, Chadwick J, Shvets E, Cowburn AS, Dunmore BJ, Crosby A, Morrell NW, Nichols BJ (2015). Caveolae protect endothelial cells from membrane rupture during increased cardiac output. The Journal of Cell Biology.

[bib6] Cheng JPX, Nichols BJ (2016). Caveolae: one function or many?. Trends in Cell Biology.

[bib7] Clarke JP, Mearow KM (2013). Cell stress promotes the association of phosphorylated HspB1 with F-actin. PLOS ONE.

[bib8] Collier MP, Alderson TR, de Villiers CP, Nicholls D, Gastall HY, Allison TM, Degiacomi MT, Jiang H, Mlynek G, Fürst DO, van der Ven PFM, Djinovic-Carugo K, Baldwin AJ, Watkins H, Gehmlich K, Benesch JLP (2019). HspB1 phosphorylation regulates its intramolecular dynamics and mechanosensitive molecular chaperone interaction with filamin C. Science Advances.

[bib9] De Belly H, Yan S, Borja da Rocha H, Ichbiah S, Town JP, Zager PJ, Estrada DC, Meyer K, Turlier H, Bustamante C, Weiner OD (2023). Cell protrusions and contractions generate long-range membrane tension propagation. Cell.

[bib10] Del Pozo MA, Lolo FN, Echarri A (2021). Caveolae: Mechanosensing and mechanotransduction devices linking membrane trafficking to mechanoadaptation. Current Opinion in Cell Biology.

[bib11] Dewulf M, Köster DV, Sinha B, Viaris de Lesegno C, Chambon V, Bigot A, Bensalah M, Negroni E, Tardif N, Podkalicka J, Johannes L, Nassoy P, Butler-Browne G, Lamaze C, Blouin CM (2019). Dystrophy-associated caveolin-3 mutations reveal that caveolae couple IL6/STAT3 signaling with mechanosensing in human muscle cells. Nature Communications.

[bib12] Echarri A, Del Pozo MA (2015). Caveolae - mechanosensitive membrane invaginations linked to actin filaments. Journal of Cell Science.

[bib13] Gambin Y, Ariotti N, McMahon K-A, Bastiani M, Sierecki E, Kovtun O, Polinkovsky ME, Magenau A, Jung W, Okano S, Zhou Y, Leneva N, Mureev S, Johnston W, Gaus K, Hancock JF, Collins BM, Alexandrov K, Parton RG (2014). Single-molecule analysis reveals self assembly and nanoscale segregation of two distinct cavin subcomplexes on caveolae. eLife.

[bib14] Garcia J, Bagwell J, Njaine B, Norman J, Levic DS, Wopat S, Miller SE, Liu X, Locasale JW, Stainier DYR, Bagnat M (2017). Sheath cell invasion and trans-differentiation repair mechanical damage caused by loss of caveolae in the Zebrafish Notochord. Current Biology.

[bib15] García-Arcos JM, Mehidi A, Velázquez JS, Guillamat P, Tomba C, Houzet L, Capolupo L, D’Angelo G, Colom A, Hinde E, Aumeier C, Roux A (2024). Actin dynamics sustains spatial gradients of membrane tension in adherent cells. bioRxiv.

[bib16] Golani G, Ariotti N, Parton RG, Kozlov MM (2019). Membrane curvature and tension control the formation and collapse of caveolar superstructures. Developmental Cell.

[bib17] Grande-García A, Echarri A, de Rooij J, Alderson NB, Waterman-Storer CM, Valdivielso JM, del Pozo MA (2007). Caveolin-1 regulates cell polarization and directional migration through Src kinase and Rho GTPases. The Journal of Cell Biology.

[bib18] Grande-García A, del Pozo MA (2008). Caveolin-1 in cell polarization and directional migration. European Journal of Cell Biology.

[bib19] Guo Y, Yang L, Haught K, Scarlata S (2015). Osmotic stress reduces Ca2+ signals through deformation of caveolae. The Journal of Biological Chemistry.

[bib20] Hansen CG, Howard G, Nichols BJ (2011). Pacsin 2 is recruited to caveolae and functions in caveolar biogenesis. Journal of Cell Science.

[bib21] Hetmanski JHR, de Belly H, Busnelli I, Waring T, Nair RV, Sokleva V, Dobre O, Cameron A, Gauthier N, Lamaze C, Swift J, Del Campo A, Starborg T, Zech T, Goetz JG, Paluch EK, Schwartz J-M, Caswell PT (2019). Membrane tension orchestrates rear retraction in matrix-directed cell migration. Developmental Cell.

[bib22] Hetmanski JHR, Jones MC, Chunara F, Schwartz JM, Caswell PT (2021). Combinatorial mathematical modelling approaches to interrogate rear retraction dynamics in 3D cell migration. PLOS Computational Biology.

[bib23] Hoffman LM, Jensen CC, Beckerle MC (2022). Phosphorylation of the small heat shock protein HspB1 regulates cytoskeletal recruitment and cell motility. Molecular Biology of the Cell.

[bib24] Hossain MM, Zhao G, Woo M-S, Wang JH-C, Jin J-P (2016). Deletion of Calponin 2 in mouse fibroblasts increases myosin II-dependent cell traction force. Biochemistry.

[bib25] Houk AR, Jilkine A, Mejean CO, Boltyanskiy R, Dufresne ER, Angenent SB, Altschuler SJ, Wu LF, Weiner OD (2012). Membrane tension maintains cell polarity by confining signals to the leading edge during neutrophil migration. Cell.

[bib26] Hung V, Udeshi ND, Lam SS, Loh KH, Cox KJ, Pedram K, Carr SA, Ting AY (2016). Spatially resolved proteomic mapping in living cells with the engineered peroxidase APEX2. Nature Protocols.

[bib27] Joshi B, Strugnell SS, Goetz JG, Kojic LD, Cox ME, Griffith OL, Chan SK, Jones SJ, Leung S-P, Masoudi H, Leung S, Wiseman SM, Nabi IR (2008). Phosphorylated caveolin-1 regulates Rho/ROCK-dependent focal adhesion dynamics and tumor cell migration and invasion. Cancer Research.

[bib28] Joshi B, Bastiani M, Strugnell SS, Boscher C, Parton RG, Nabi IR (2012). Phosphocaveolin-1 is a mechanotransducer that induces caveola biogenesis via Egr1 transcriptional regulation. The Journal of Cell Biology.

[bib29] Kanai Y, Wang D, Hirokawa N (2014). KIF13B enhances the endocytosis of LRP1 by recruiting LRP1 to caveolae. The Journal of Cell Biology.

[bib30] Kovtun O, Tillu VA, Jung W, Leneva N, Ariotti N, Chaudhary N, Mandyam RA, Ferguson C, Morgan GP, Johnston WA, Harrop SJ, Alexandrov K, Parton RG, Collins BM (2014). Structural insights into the organization of the cavin membrane coat complex. Developmental Cell.

[bib31] Kovtun O, Tillu VA, Ariotti N, Parton RG, Collins BM (2015). Cavin family proteins and the assembly of caveolae. Journal of Cell Science.

[bib32] Lam SS, Martell JD, Kamer KJ, Deerinck TJ, Ellisman MH, Mootha VK, Ting AY (2015). Directed evolution of APEX2 for electron microscopy and proximity labeling. Nature Methods.

[bib33] Li H, Hou S, Wu X, Nandagopal S, Lin F, Kung S, Marshall AJ (2013). The Tandem PH domain-containing protein 2 (TAPP2) regulates chemokine-induced cytoskeletal reorganization and malignant B cell migration. PLOS ONE.

[bib34] Lieber AD, Yehudai-Resheff S, Barnhart EL, Theriot JA, Keren K (2013). Membrane tension in rapidly moving cells is determined by cytoskeletal forces. Current Biology.

[bib35] Lieber AD, Schweitzer Y, Kozlov MM, Keren K (2015). Front-to-rear membrane tension gradient in rapidly moving cells. Biophysical Journal.

[bib36] Lim Y-W, Lo HP, Ferguson C, Martel N, Giacomotto J, Gomez GA, Yap AS, Hall TE, Parton RG (2017). Caveolae protect notochord cells against catastrophic mechanical failure during development. Current Biology.

[bib37] Liu R, Jin JP (2016). Calponin isoforms CNN1, CNN2 and CNN3: regulators for actin cytoskeleton functions in smooth muscle and non-muscle cells. Gene.

[bib38] Lo HP, Nixon SJ, Hall TE, Cowling BS, Ferguson C, Morgan GP, Schieber NL, Fernandez-Rojo MA, Bastiani M, Floetenmeyer M, Martel N, Laporte J, Pilch PF, Parton RG (2015). The caveolin-cavin system plays a conserved and critical role in mechanoprotection of skeletal muscle. The Journal of Cell Biology.

[bib39] Lolo F-N, Pavón DM, Grande-García A, Elosegui-Artola A, Segatori VI, Sánchez S, Trepat X, Roca-Cusachs P, Del Pozo MA (2022). Caveolae couple mechanical stress to integrin recycling and activation. eLife.

[bib40] Lolo F-N, Walani N, Seemann E, Zalvidea D, Pavón DM, Cojoc G, Zamai M, Viaris de Lesegno C, Martínez de Benito F, Sánchez-Álvarez M, Uriarte JJ, Echarri A, Jiménez-Carretero D, Escolano J-C, Sánchez SA, Caiolfa VR, Navajas D, Trepat X, Guck J, Lamaze C, Roca-Cusachs P, Kessels MM, Qualmann B, Arroyo M, Del Pozo MA (2023). Caveolin-1 dolines form a distinct and rapid caveolae-independent mechanoadaptation system. Nature Cell Biology.

[bib41] Lu T, Wang XL, Chai Q, Sun X, Sieck GC, Katusic ZS, Lee HC (2017). Role of the endothelial caveolae microdomain in shear stress-mediated coronary vasorelaxation. The Journal of Biological Chemistry.

[bib42] Ludwig A, Howard G, Mendoza-Topaz C, Deerinck T, Mackey M, Sandin S, Ellisman MH, Nichols BJ (2013). Molecular composition and ultrastructure of the caveolar coat complex. PLOS Biology.

[bib43] Ludwig A, Nichols BJ, Sandin S (2016). Architecture of the caveolar coat complex. Journal of Cell Science.

[bib44] Ludwig A, Nguyen TH, Leong D, Ravi LI, Tan BH, Sandin S, Sugrue RJ (2017). Caveolae provide a specialized membrane environment for respiratory syncytial virus assembly. Journal of Cell Science.

[bib45] Ludwig A (2020). Selective visualization of caveolae by TEM Using APEX2. Methods in Molecular Biology.

[bib46] Matthaeus C, Sochacki KA, Dickey AM, Puchkov D, Haucke V, Lehmann M, Taraska JW (2022). The molecular organization of differentially curved caveolae indicates bendable structural units at the plasma membrane. Nature Communications.

[bib47] Mayanagi T, Sobue K (2011). Diversification of caldesmon-linked actin cytoskeleton in cell motility. Cell Adhesion & Migration.

[bib48] McMahon K-A, Wu Y, Gambin Y, Sierecki E, Tillu VA, Hall T, Martel N, Okano S, Moradi SV, Ruelcke JE, Ferguson C, Yap AS, Alexandrov K, Hill MM, Parton RG (2019). Identification of intracellular cavin target proteins reveals cavin-PP1alpha interactions regulate apoptosis. Nature Communications.

[bib49] McMahon K-A, Stroud DA, Gambin Y, Tillu V, Bastiani M, Sierecki E, Polinkovsky ME, Hall TE, Gomez GA, Wu Y, Parat M-O, Martel N, Lo HP, Khanna KK, Alexandrov K, Daly R, Yap A, Ryan MT, Parton RG (2021). Cavin3 released from caveolae interacts with BRCA1 to regulate the cellular stress response. eLife.

[bib50] Mendoza-Topaz C, Yeow I, Riento K, Nichols BJ (2018). BioID identifies proteins involved in the cell biology of caveolae. PLOS ONE.

[bib51] Meng F, Saxena S, Liu Y, Joshi B, Wong TH, Shankar J, Foster LJ, Bernatchez P, Nabi IR (2017). The phospho-caveolin-1 scaffolding domain dampens force fluctuations in focal adhesions and promotes cancer cell migration. Molecular Biology of the Cell.

[bib52] Meng Z, Qiu Y, Lin KC, Kumar A, Placone JK, Fang C, Wang K-C, Lu S, Pan M, Hong AW, Moroishi T, Luo M, Plouffe SW, Diao Y, Ye Z, Park HW, Wang X, Yu F-X, Chien S, Wang C-Y, Ren B, Engler AJ, Guan K-L (2018). RAP2 mediates mechanoresponses of the Hippo pathway. Nature.

[bib53] Morén B, Shah C, Howes MT, Schieber NL, McMahon HT, Parton RG, Daumke O, Lundmark R (2012). EHD2 regulates caveolar dynamics via ATP-driven targeting and oligomerization. Molecular Biology of the Cell.

[bib54] Moreno-Vicente R, Pavón DM, Martín-Padura I, Català-Montoro M, Díez-Sánchez A, Quílez-Álvarez A, López JA, Sánchez-Álvarez M, Vázquez J, Strippoli R, Del Pozo MA (2019). Caveolin-1 modulates mechanotransduction responses to substrate stiffness through actin-dependent control of YAP. Cell Reports.

[bib55] Mougeolle A, Poussard S, Decossas M, Lamaze C, Lambert O, Dargelos E (2015). Oxidative stress induces caveolin 1 degradation and impairs caveolae functions in skeletal muscle cells. PLOS ONE.

[bib56] Mseka T, Cramer LP (2011). Actin depolymerization-based force retracts the cell rear in polarizing and migrating cells. Current Biology.

[bib57] Mueller J, Szep G, Nemethova M, de Vries I, Lieber AD, Winkler C, Kruse K, Small JV, Schmeiser C, Keren K, Hauschild R, Sixt M (2017). Load Adaptation of lamellipodial actin networks. Cell.

[bib58] Muriel O, Echarri A, Hellriegel C, Pavón DM, Beccari L, Del Pozo MA (2011). Phosphorylated filamin A regulates actin-linked caveolae dynamics. Journal of Cell Science.

[bib59] Nassoy P, Lamaze C (2012). Stressing caveolae new role in cell mechanics. Trends in Cell Biology.

[bib60] Nethe M, Hordijk PL (2011). A model for phospho-caveolin-1-driven turnover of focal adhesions. Cell Adhesion & Migration.

[bib61] Palma-Flores C, Ramírez-Sánchez I, Rosas-Vargas H, Canto P, Coral-Vázquez RM (2014). Description of a utrophin associated protein complex in lipid raft domains of human artery smooth muscle cells. Biochimica et Biophysica Acta.

[bib62] Parton RG, Simons K (2007). The multiple faces of caveolae. Nature Reviews. Molecular Cell Biology.

[bib63] Parton RG, del Pozo MA (2013). Caveolae as plasma membrane sensors, protectors and organizers. Nature Reviews. Molecular Cell Biology.

[bib64] Parton RG, McMahon KA, Wu Y (2020). Caveolae: formation, dynamics, and function. Current Opinion in Cell Biology.

[bib65] Post A, Pannekoek WJ, Ponsioen B, Vliem MJ, Bos JL (2015). Rap1 spatially controls ArhGAP29 To inhibit rho signaling during endothelial barrier regulation. Molecular and Cellular Biology.

[bib66] Qiao Y, Chen J, Lim YB, Finch-Edmondson ML, Seshachalam VP, Qin L, Jiang T, Low BC, Singh H, Lim CT, Sudol M (2017). YAP regulates actin dynamics through ARHGAP29 and promotes metastasis. Cell Reports.

[bib67] Rajaganapathy S, McCourt JL, Ghosal S, Lindsay A, McCourt PM, Lowe DA, Ervasti JM, Salapaka MV (2019). Distinct mechanical properties in homologous spectrin-like repeats of utrophin. Scientific Reports.

[bib68] Rausch V, Bostrom JR, Park J, Bravo IR, Feng Y, Hay DC, Link BA, Hansen CG (2019). The hippo pathway regulates caveolae expression and mediates flow response via caveolae. Current Biology.

[bib69] Richter T, Floetenmeyer M, Ferguson C, Galea J, Goh J, Lindsay MR, Morgan GP, Marsh BJ, Parton RG (2008). High-resolution 3D quantitative analysis of caveolar ultrastructure and caveola-cytoskeleton interactions. Traffic.

[bib70] Ridley AJ, Schwartz MA, Burridge K, Firtel RA, Ginsberg MH, Borisy G, Parsons JT, Horwitz AR (2003). Cell migration: integrating signals from front to back. Science.

[bib71] Rogg M, Maier JI, Helmstädter M, Sammarco A, Kliewe F, Kretz O, Weißer L, Van Wymersch C, Findeisen K, Koessinger AL, Tsoy O, Baumbach J, Grabbert M, Werner M, Huber TB, Endlich N, Schilling O, Schell C (2023). A YAP/TAZ-ARHGAP29-RhoA Signaling axis regulates podocyte protrusions and integrin adhesions. Cells.

[bib72] Schindelin J, Arganda-Carreras I, Frise E, Kaynig V, Longair M, Pietzsch T, Preibisch S, Rueden C, Saalfeld S, Schmid B, Tinevez J-Y, White DJ, Hartenstein V, Eliceiri K, Tomancak P, Cardona A (2012). Fiji: an open-source platform for biological-image analysis. Nature Methods.

[bib73] Schneider CA, Rasband WS, Eliceiri KW (2012). NIH Image to ImageJ: 25 years of image analysis. Nature Methods.

[bib74] Senju Y, Itoh Y, Takano K, Hamada S, Suetsugu S (2011). Essential role of PACSIN2/syndapin-II in caveolae membrane sculpting. Journal of Cell Science.

[bib75] Shi Z, Graber ZT, Baumgart T, Stone HA, Cohen AE (2018). Cell membranes resist flow. Cell.

[bib76] Shi X, Wen Z, Wang Y, Liu YJ, Shi K, Jiu Y (2021). Feedback-driven mechanisms between phosphorylated caveolin-1 and Contractile actin assemblies instruct persistent cell migration. Frontiers in Cell and Developmental Biology.

[bib77] Shu X, Lev-Ram V, Deerinck TJ, Qi Y, Ramko EB, Davidson MW, Jin Y, Ellisman MH, Tsien RY (2011). A genetically encoded tag for correlated light and electron microscopy of intact cells, tissues, and organisms. PLOS Biology.

[bib78] Shvets E, Ludwig A, Nichols BJ (2014). News from the caves: update on the structure and function of caveolae. Current Opinion in Cell Biology.

[bib79] Sinha B, Köster D, Ruez R, Gonnord P, Bastiani M, Abankwa D, Stan RV, Butler-Browne G, Vedie B, Johannes L, Morone N, Parton RG, Raposo G, Sens P, Lamaze C, Nassoy P (2011). Cells respond to mechanical stress by rapid disassembly of caveolae. Cell.

[bib80] Sitarska E, Diz-Muñoz A (2020). Pay attention to membrane tension: mechanobiology of the cell surface. Current Opinion in Cell Biology.

[bib81] Sobue K, Sellers JR (1991). Caldesmon, a novel regulatory protein in smooth muscle and nonmuscle actomyosin systems. The Journal of Biological Chemistry.

[bib82] Stahlhut M, van Deurs B (2000). Identification of filamin as a novel ligand for caveolin-1: evidence for the organization of caveolin-1-associated membrane domains by the actin cytoskeleton. Molecular Biology of the Cell.

[bib83] Stoeber M, Stoeck IK, Hänni C, Bleck CKE, Balistreri G, Helenius A (2012). Oligomers of the ATPase EHD2 confine caveolae to the plasma membrane through association with actin. The EMBO Journal.

[bib84] Stoeber M, Schellenberger P, Siebert CA, Leyrat C, Helenius A, Grünewald K (2016). Model for the architecture of caveolae based on a flexible, net-like assembly of Cavin1 and Caveolin discs. PNAS.

[bib85] Stossel TP, Condeelis J, Cooley L, Hartwig JH, Noegel A, Schleicher M, Shapiro SS (2001). Filamins as integrators of cell mechanics and signalling. Nature Reviews. Molecular Cell Biology.

[bib86] Sverdlov M, Shinin V, Place AT, Castellon M, Minshall RD (2009). Filamin A regulates caveolae internalization and trafficking in endothelial cells. Molecular Biology of the Cell.

[bib87] Tan PY, Zaidel-Bar R (2015). Transient membrane localization of SPV-1 drives cyclical actomyosin contractions in the *C. elegans* spermatheca. Current Biology.

[bib88] Tan B, Peng S, Yatim SMJM, Gunaratne J, Hunziker W, Ludwig A (2020a). An optimized protocol for proximity biotinylation in confluent epithelial cell cultures using the peroxidase APEX2. STAR Protocols.

[bib89] Tan B, Yatim S, Peng S, Gunaratne J, Hunziker W, Ludwig A (2020b). The mammalian crumbs complex defines a distinct polarity domain apical of epithelial tight junctions. Current Biology.

[bib90] Torrino S, Shen W-W, Blouin CM, Mani SK, Viaris de Lesegno C, Bost P, Grassart A, Köster D, Valades-Cruz CA, Chambon V, Johannes L, Pierobon P, Soumelis V, Coirault C, Vassilopoulos S, Lamaze C (2018). EHD2 is a mechanotransducer connecting caveolae dynamics with gene transcription. The Journal of Cell Biology.

[bib91] Tsai TY-C, Collins SR, Chan CK, Hadjitheodorou A, Lam P-Y, Lou SS, Yang HW, Jorgensen J, Ellett F, Irimia D, Davidson MW, Fischer RS, Huttenlocher A, Meyer T, Ferrell JE, Theriot JA (2019). Efficient front-rear coupling in neutrophil chemotaxis by Dynamic Myosin II localization. Developmental Cell.

[bib92] Ulmer B, Hagenlocher C, Schmalholz S, Kurz S, Schweickert A, Kohl A, Roth L, Sela-Donenfeld D, Blum M (2013). Calponin 2 acts as an effector of noncanonical Wnt-mediated cell polarization during neural crest cell migration. Cell Reports.

[bib93] Vaidžiulytė K, Macé A-S, Battistella A, Beng W, Schauer K, Coppey M (2022). Persistent cell migration emerges from a coupling between protrusion dynamics and polarized trafficking. eLife.

[bib94] Wiggan O, Shaw AE, DeLuca JG, Bamburg JR (2012). ADF/cofilin regulates actomyosin assembly through competitive inhibition of myosin II binding to F-actin. Developmental Cell.

[bib95] Wortel IMN, Liu AY, Dannenberg K, Berry JC, Miller MJ, Textor J (2021). CelltrackR: An R package for fast and flexible analysis of immune cell migration data. Immunoinformatics.

[bib96] Yeow I, Howard G, Chadwick J, Mendoza-Topaz C, Hansen CG, Nichols BJ, Shvets E (2017). EHD proteins cooperate to generate caveolar clusters and to maintain caveolae during repeated mechanical stress. Current Biology.

[bib97] Zimnicka AM, Husain YS, Shajahan AN, Sverdlov M, Chaga O, Chen Z, Toth PT, Klomp J, Karginov AV, Tiruppathi C, Malik AB, Minshall RD (2016). Src-dependent phosphorylation of caveolin-1 Tyr-14 promotes swelling and release of caveolae. Molecular Biology of the Cell.

