# Supplementary figures and images for: Time-resolved proximity proteomics uncovers a membrane tension-sensitive caveolin-1 interactome at the rear of migrating cells
Source: eLife. 2024 Sep 24;13:e85601. doi: 10.7554/eLife.85601 (PMC11509677; doi:10.7554/eLife.85601)

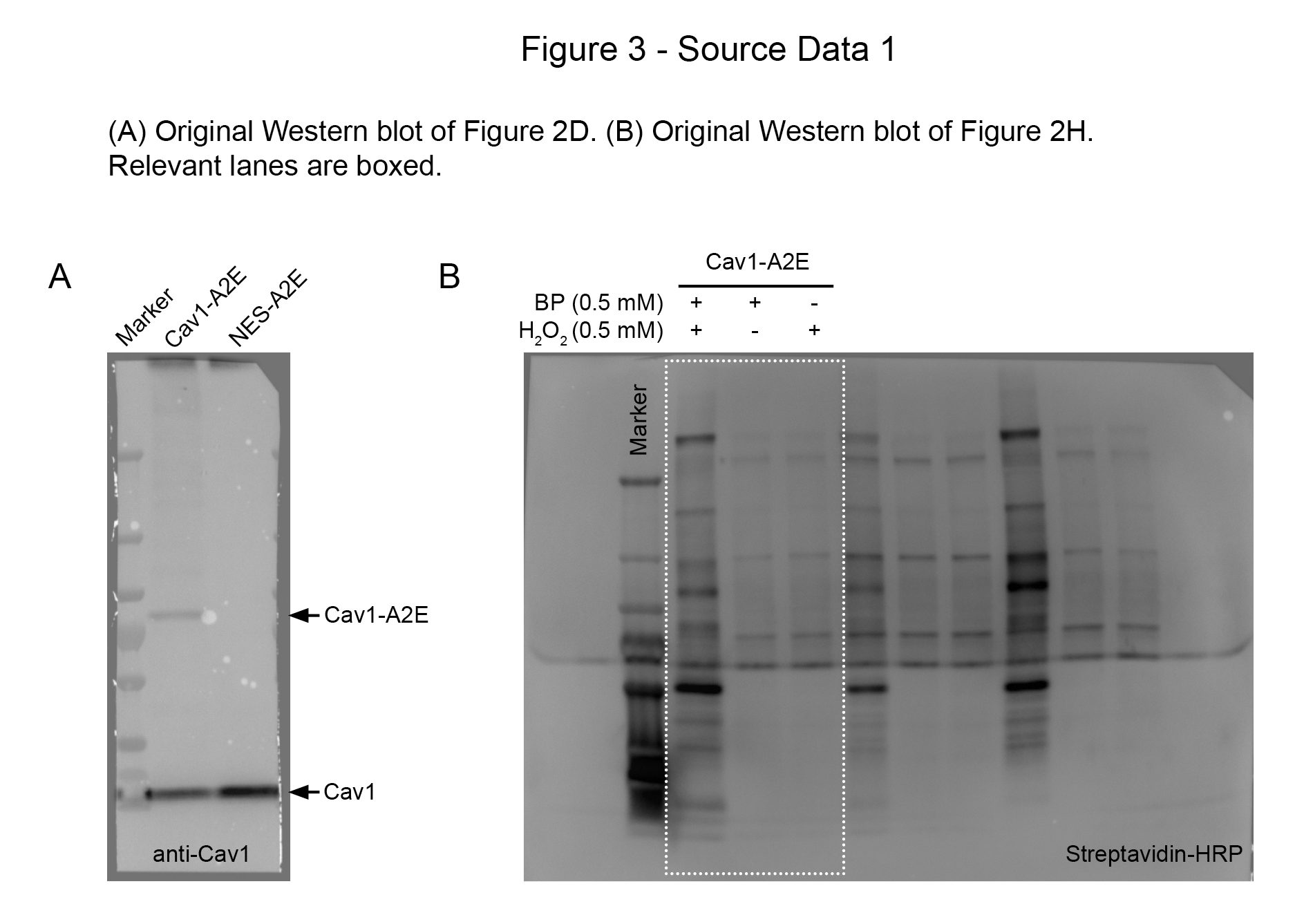

Supplement: Figure 3—source data 1. [file elife-85601-fig3-data1.zip › Figure 3 - Source Data 1/Figure 3 - Source Data 1.tif]

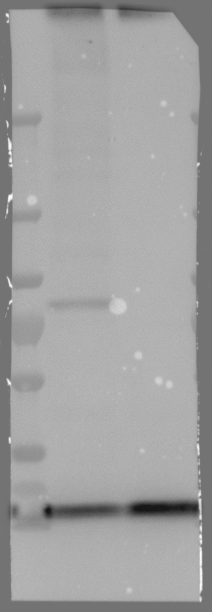

Supplement: Figure 3—source data 1. [file elife-85601-fig3-data1.zip › Figure 3 - Source Data 1/originalWB_Fig3D.Tif]

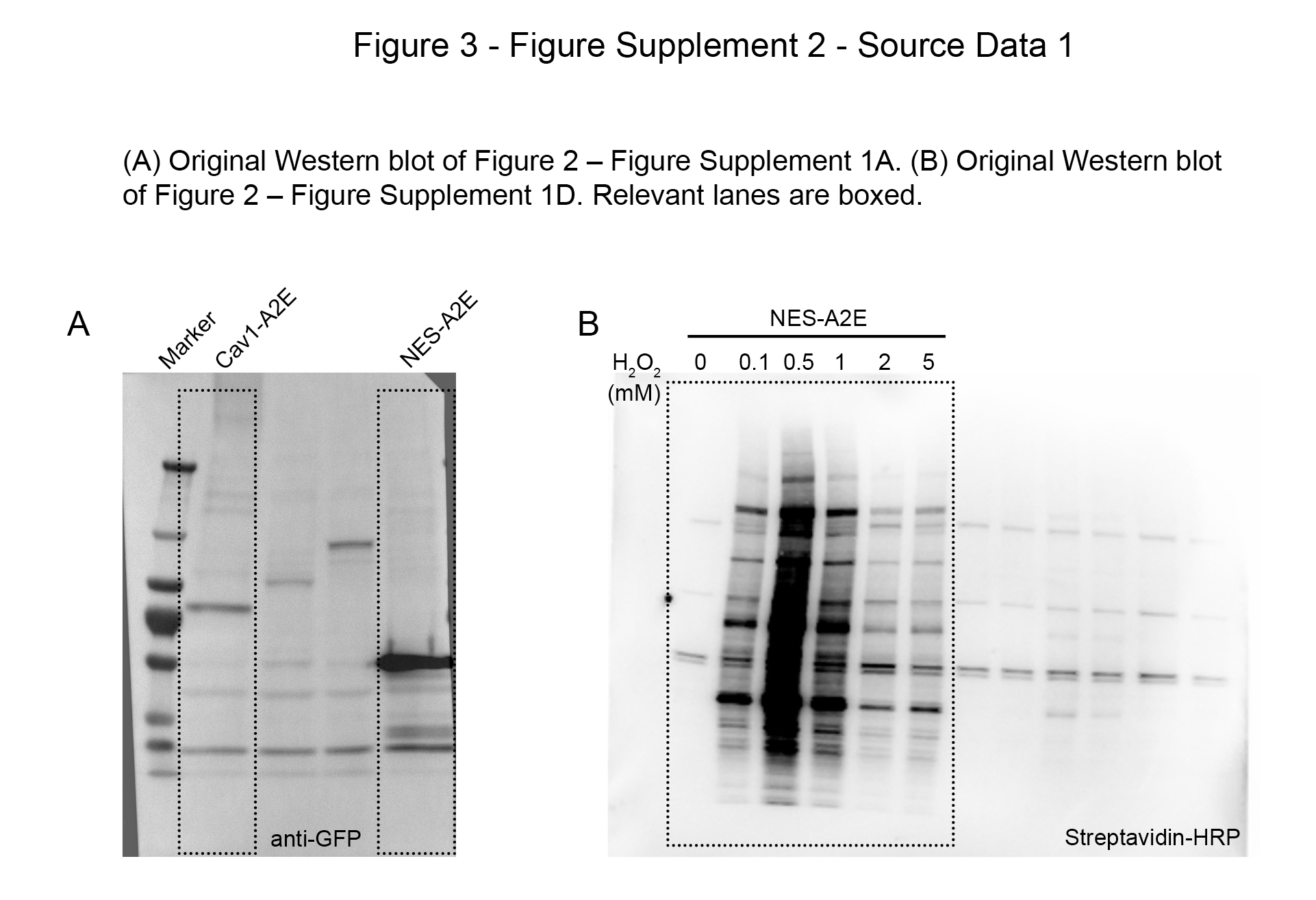

Supplement: Figure 3—figure supplement 1—source data 1. [file elife-85601-fig3-figsupp1-data1.zip › Figure 3 - Figure Supplement 1 - Source Data 1/Figure 3 - Figure Supplement 1 - Source Data 1.tif]

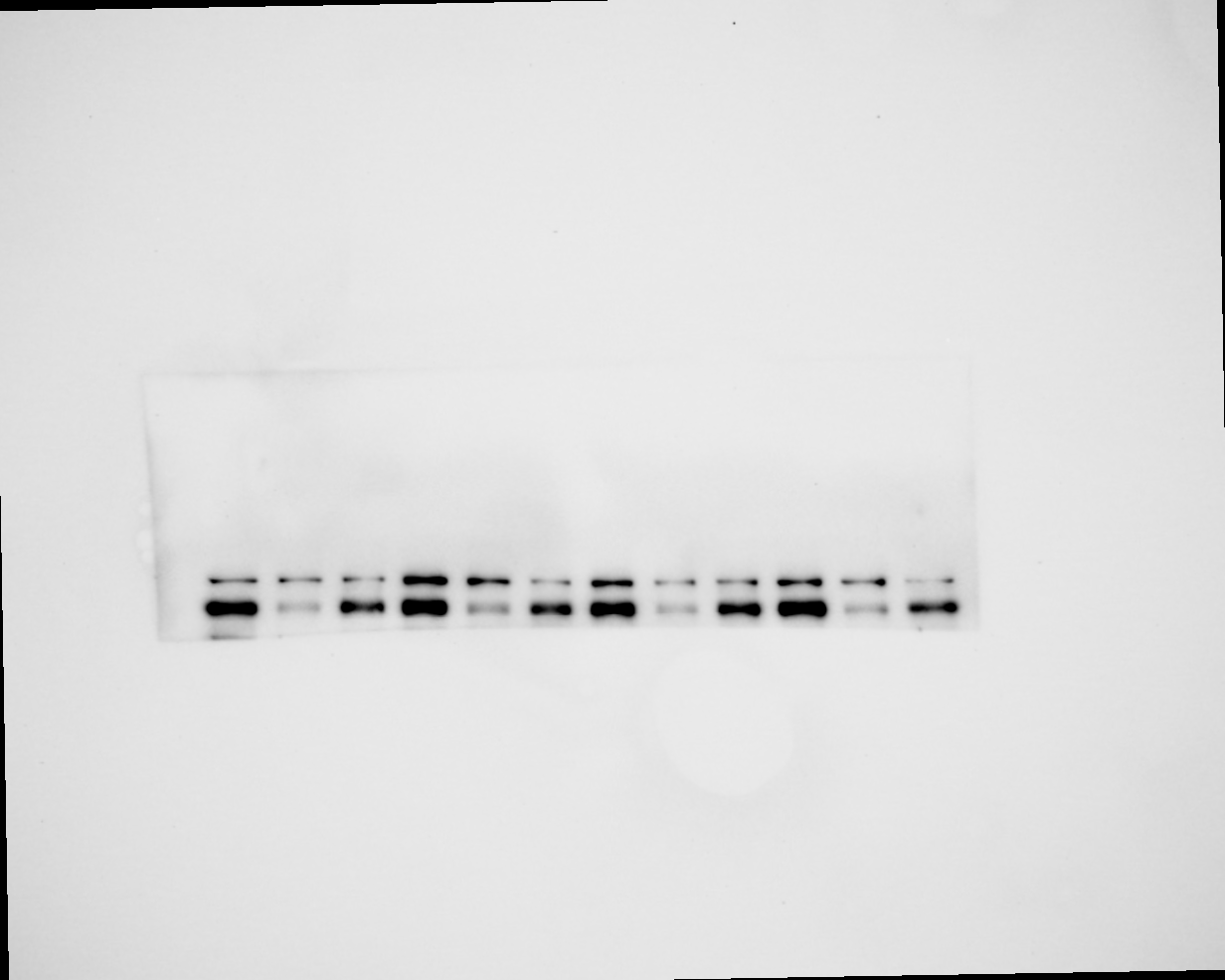

Supplement: Figure 6—source data 1. [file elife-85601-fig6-data1.zip › Figure 6 - Source Data 1/Original_Fig6A_ARHGAP29.tif]

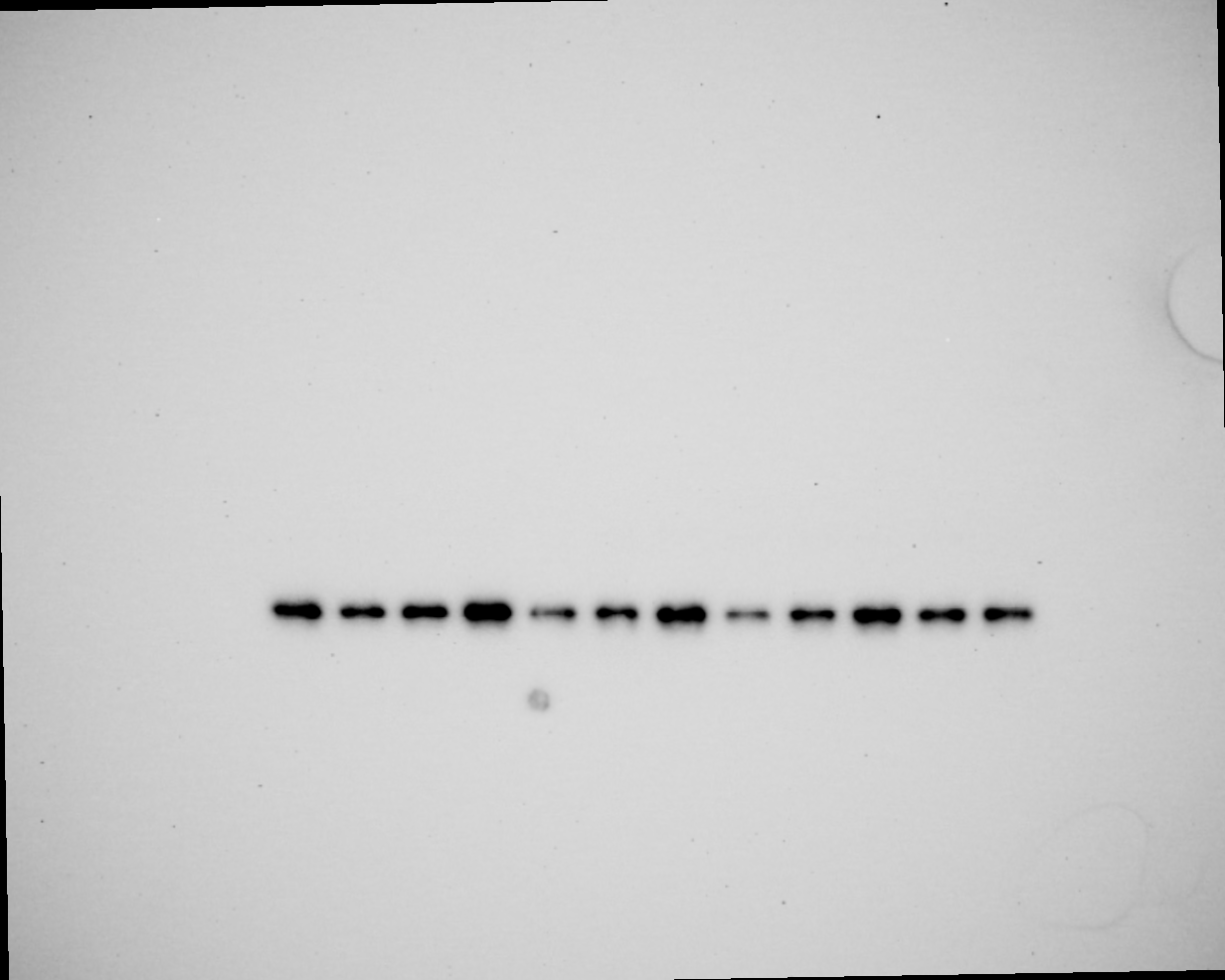

Supplement: Figure 6—source data 1. [file elife-85601-fig6-data1.zip › Figure 6 - Source Data 1/Original_Fig6A_pYAP.tif]

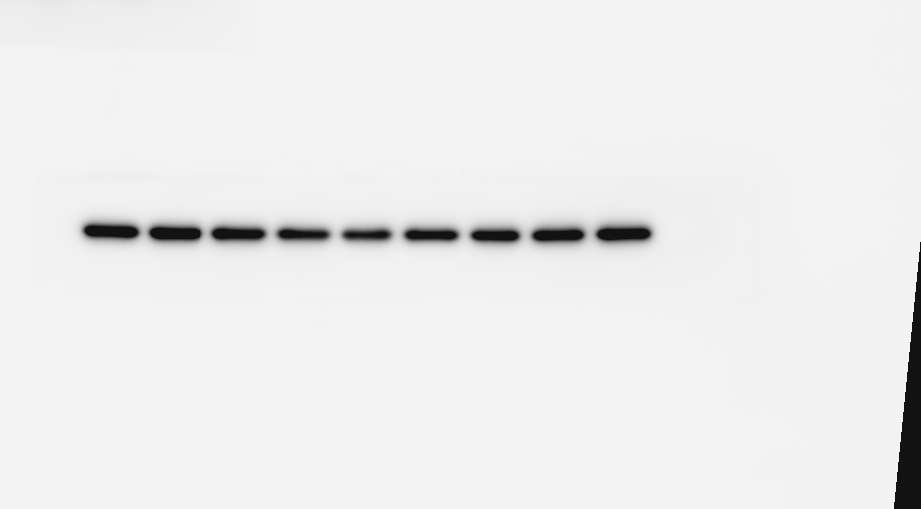

Supplement: Figure 6—source data 1. [file elife-85601-fig6-data1.zip › Figure 6 - Source Data 1/Original_Fig6A_GAPDH_blot3.tif]

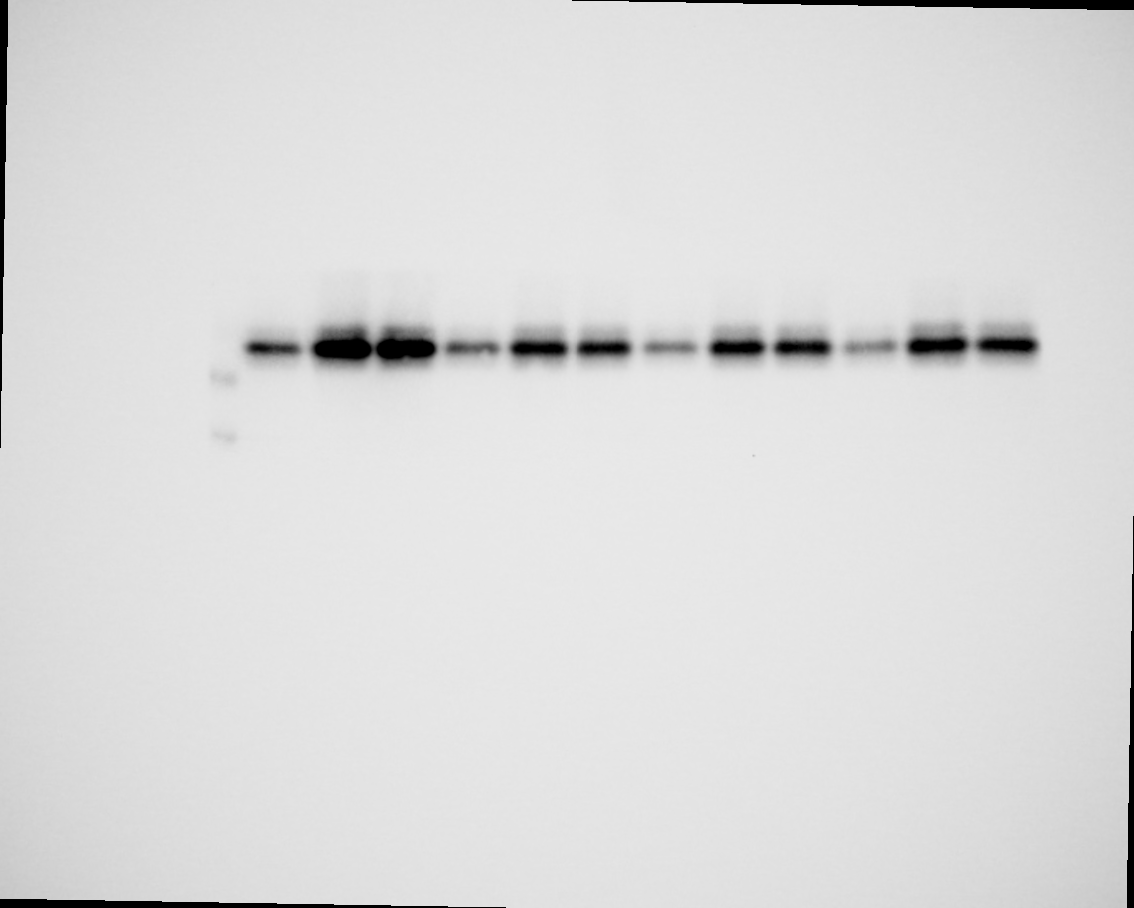

Supplement: Figure 6—source data 1. [file elife-85601-fig6-data1.zip › Figure 6 - Source Data 1/Original_Fig6A_Cav1.tif]

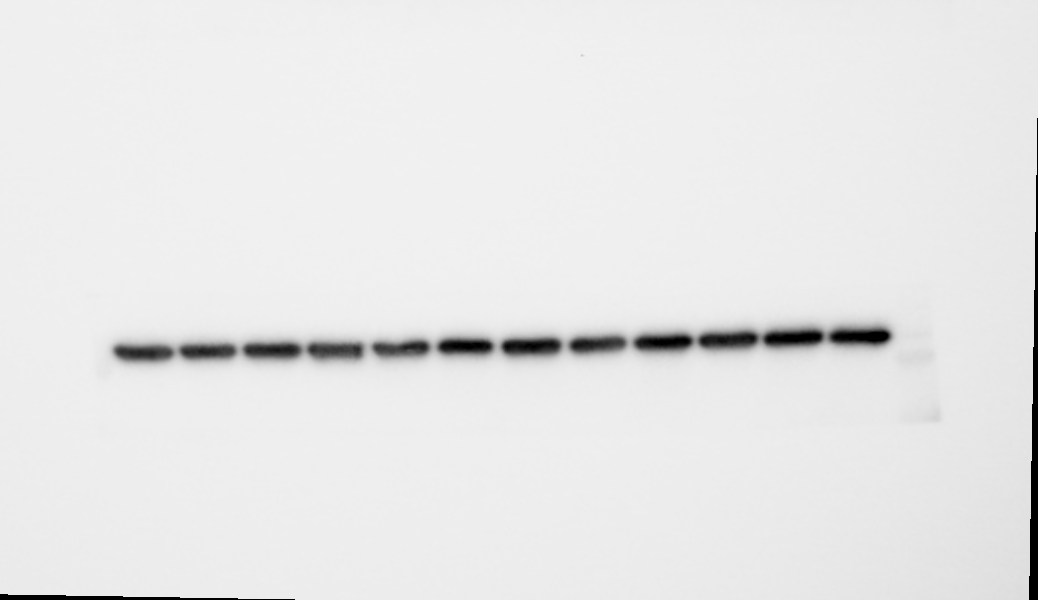

Supplement: Figure 6—source data 1. [file elife-85601-fig6-data1.zip › Figure 6 - Source Data 1/Original_Fig6A_GAPDH_blot2.tif]

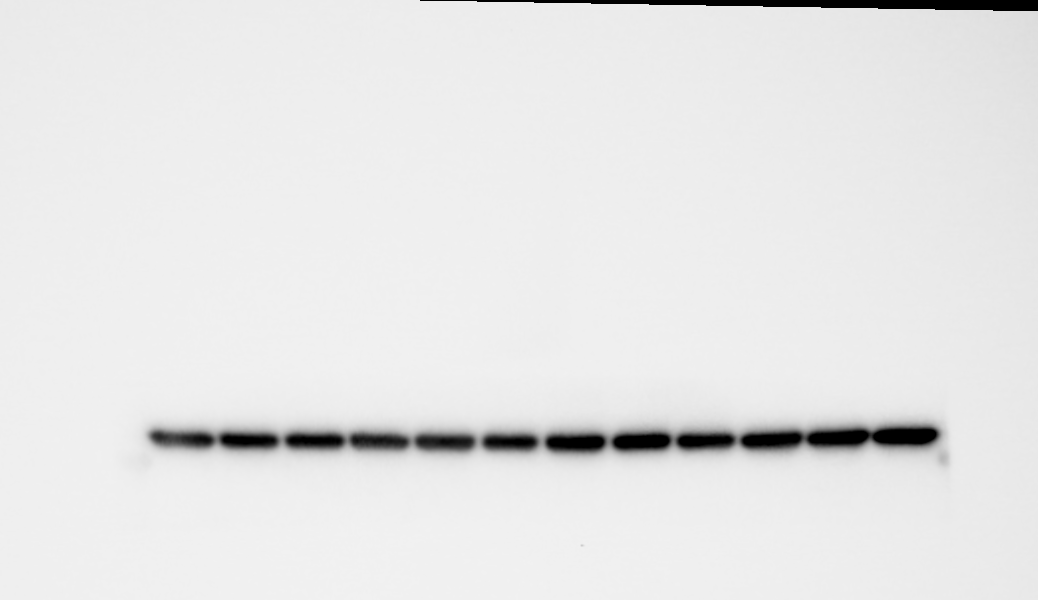

Supplement: Figure 6—source data 1. [file elife-85601-fig6-data1.zip › Figure 6 - Source Data 1/Original_Fig6A_GAPDH_blot1.tif]

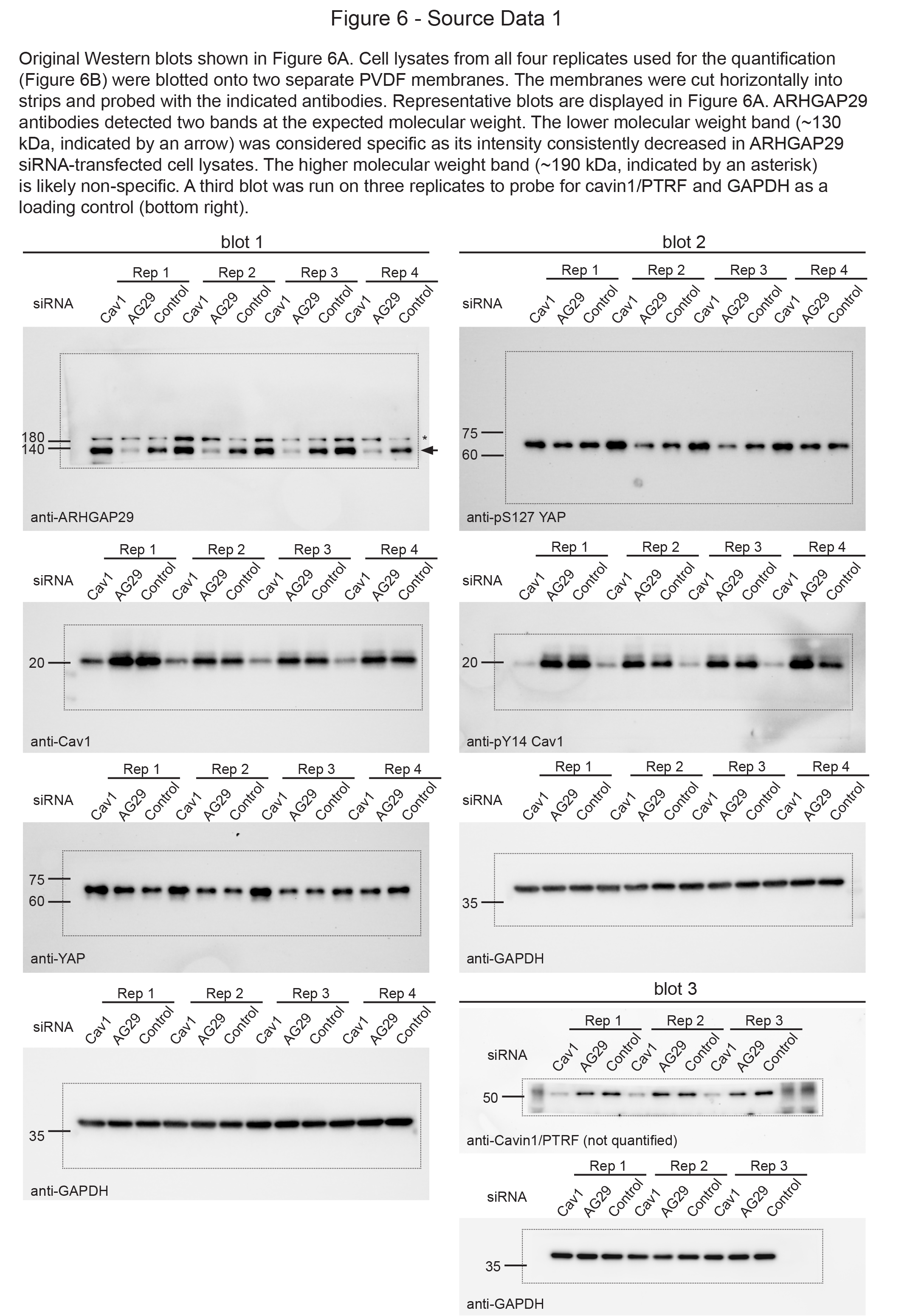

Supplement: Figure 6—source data 1. [file elife-85601-fig6-data1.zip › Figure 6 - Source Data 1/Figure 6 - Source Data 1.tif]

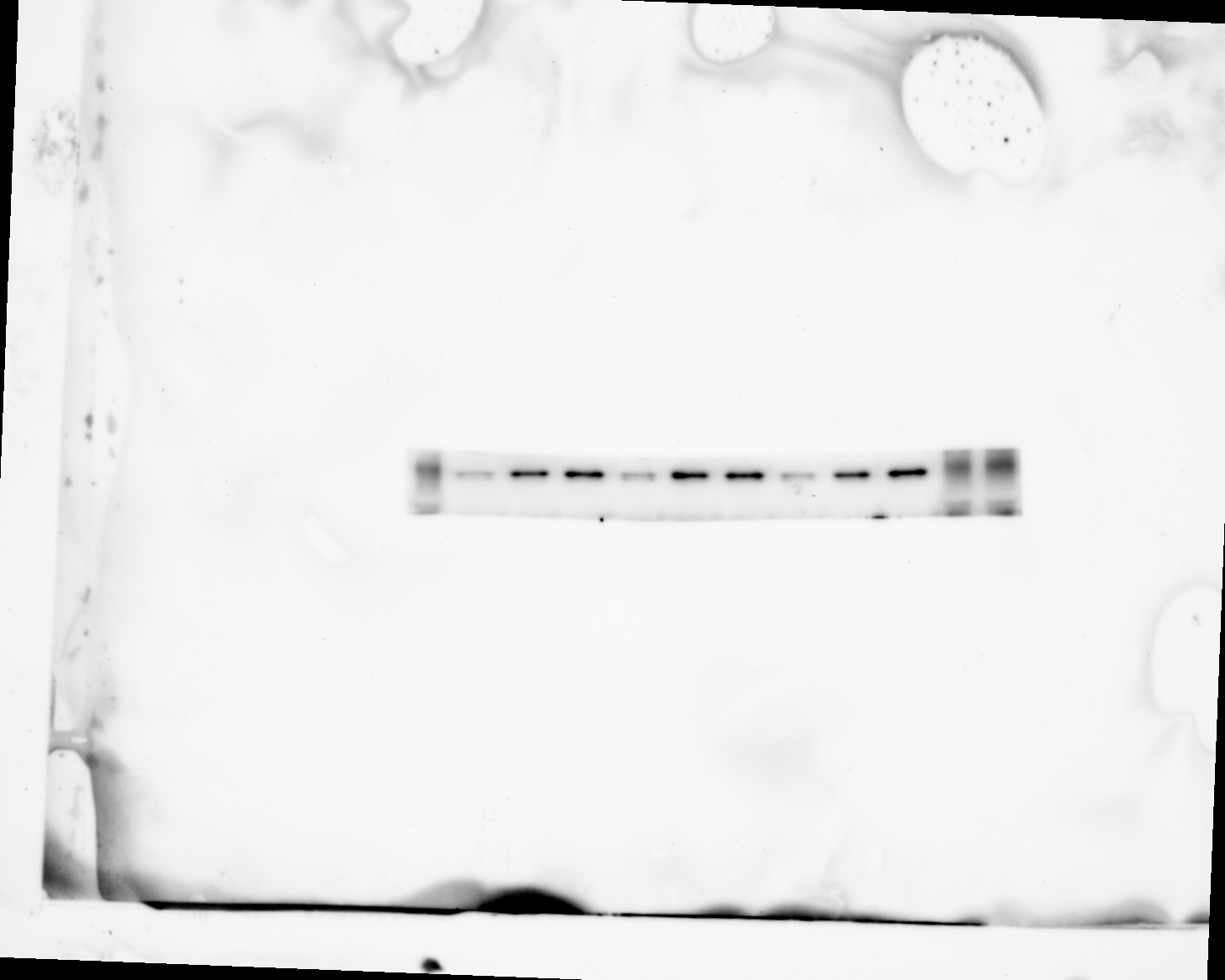

Supplement: Figure 6—source data 1. [file elife-85601-fig6-data1.zip › Figure 6 - Source Data 1/Original_Fig6A_Cavin1.tif]

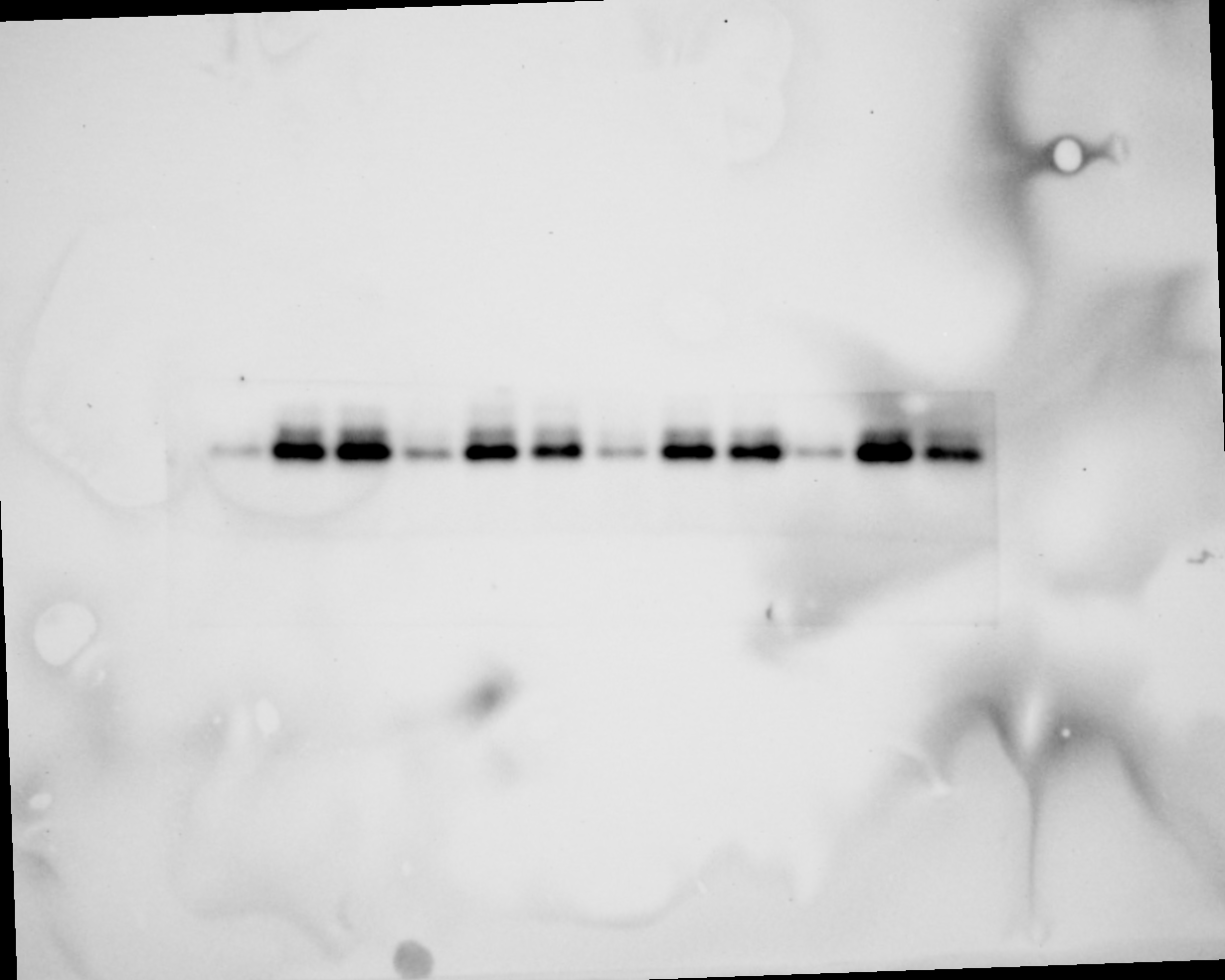

Supplement: Figure 6—source data 1. [file elife-85601-fig6-data1.zip › Figure 6 - Source Data 1/Original_Fig6A_pCav1.tif]

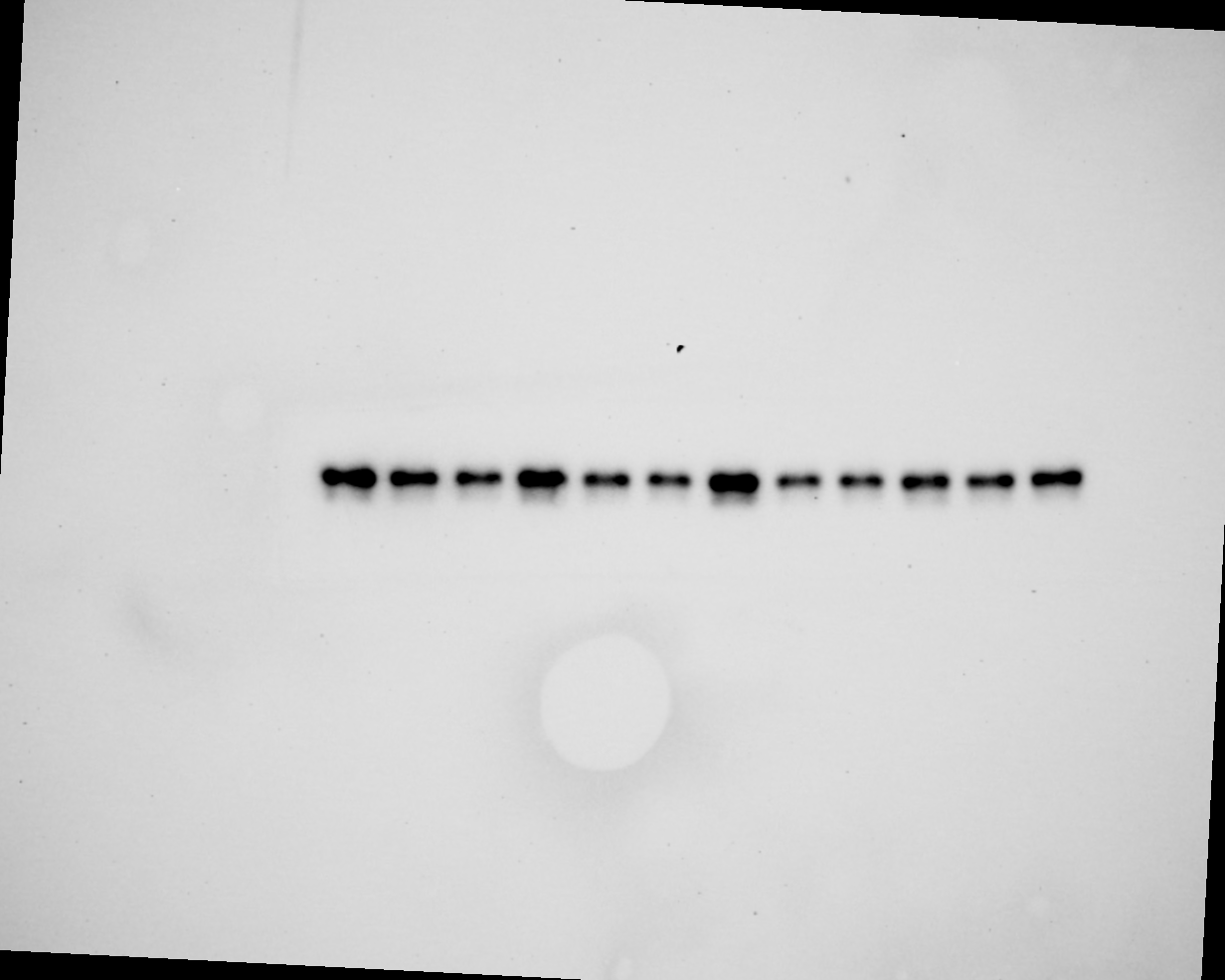

Supplement: Figure 6—source data 1. [file elife-85601-fig6-data1.zip › Figure 6 - Source Data 1/Original_Fig6A_YAP.tif]

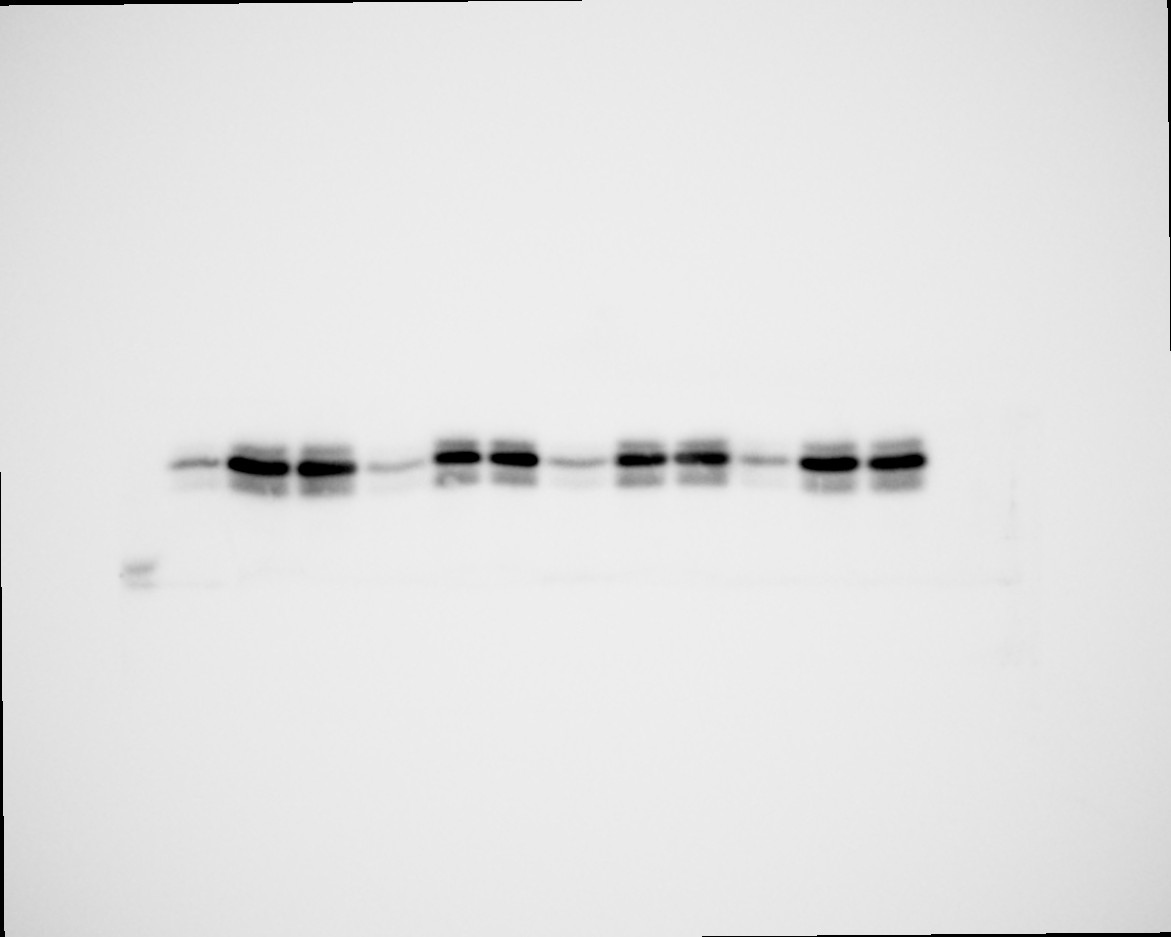

Supplement: Figure 6—source data 2. [file elife-85601-fig6-data2.zip › Figure 6 - Source Data 2/Original_pMLCquantificationFig6A_Cav1.tif]

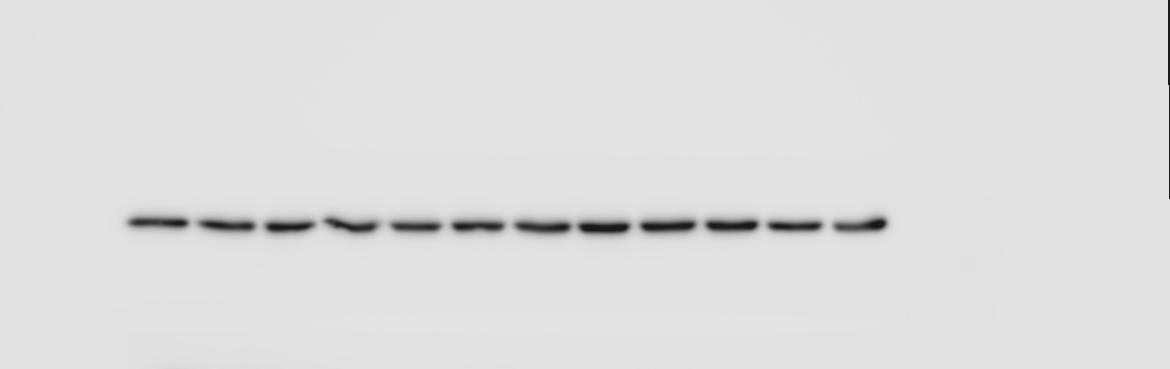

Supplement: Figure 6—source data 2. [file elife-85601-fig6-data2.zip › Figure 6 - Source Data 2/Original_pMLCquantificationFig6A_GAPDH_blot1.tif]

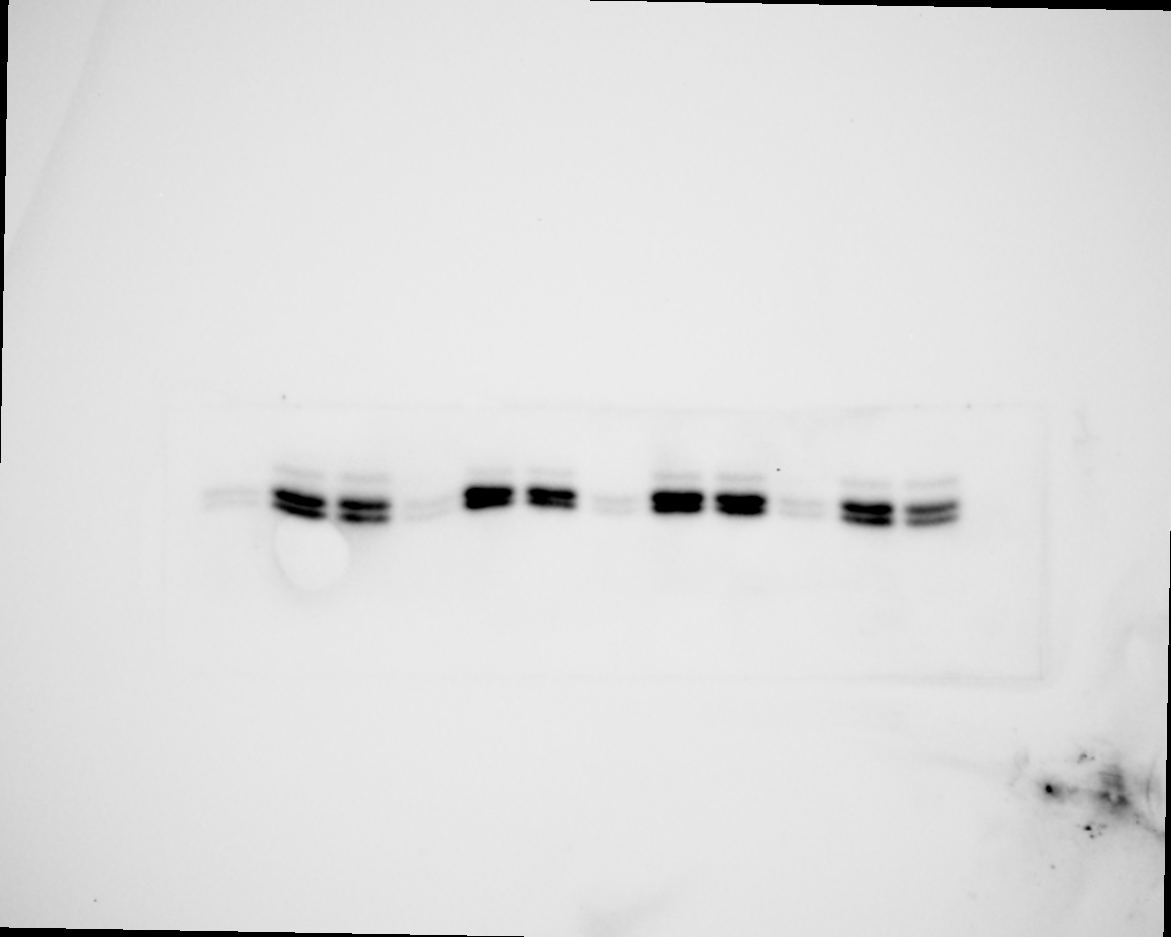

Supplement: Figure 6—source data 2. [file elife-85601-fig6-data2.zip › Figure 6 - Source Data 2/Original_pMLCquantificationFig6A_pMLC.tif]

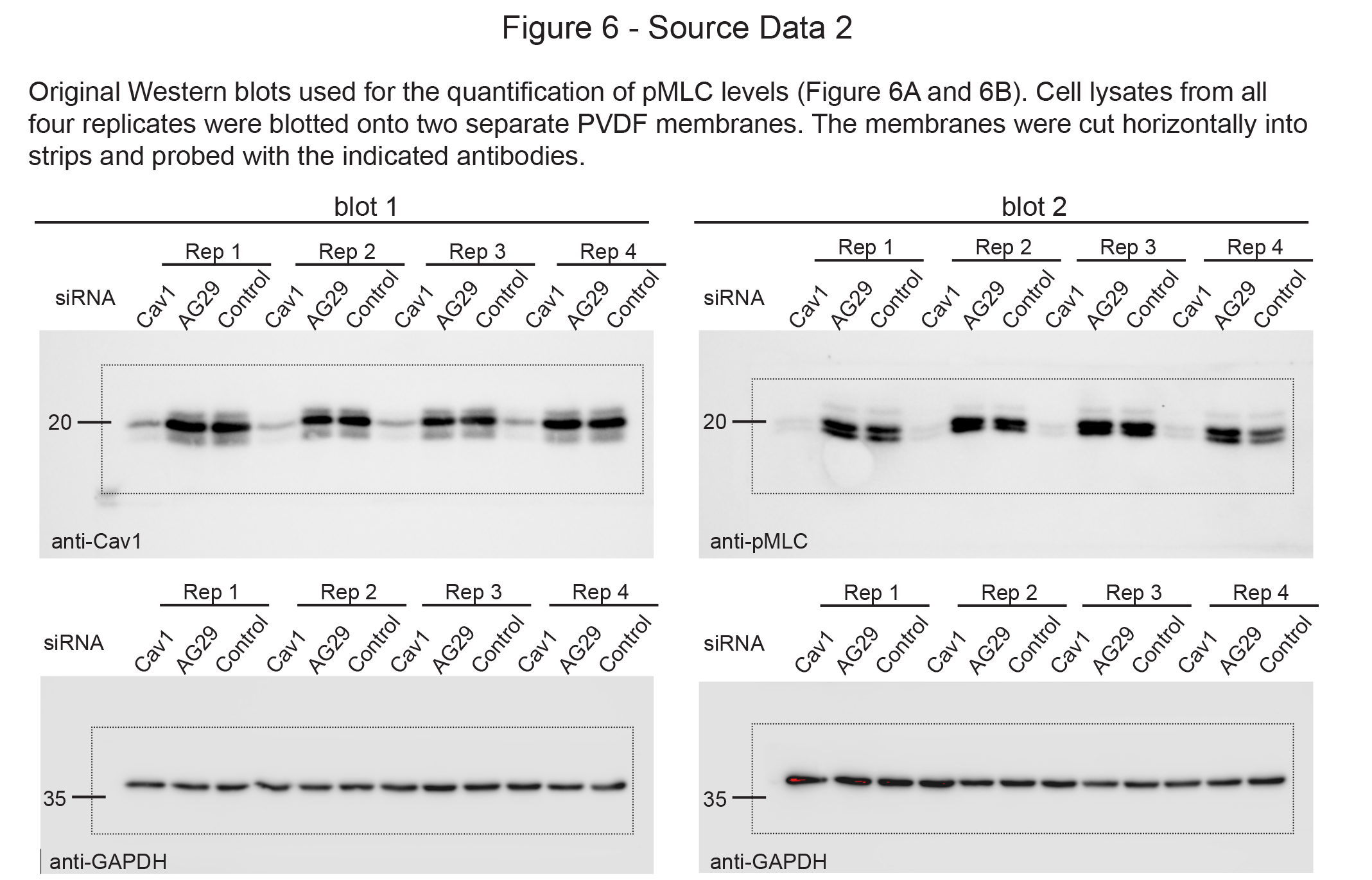

Supplement: Figure 6—source data 2. [file elife-85601-fig6-data2.zip › Figure 6 - Source Data 2/Figure 6 - Source Data 2.tif]

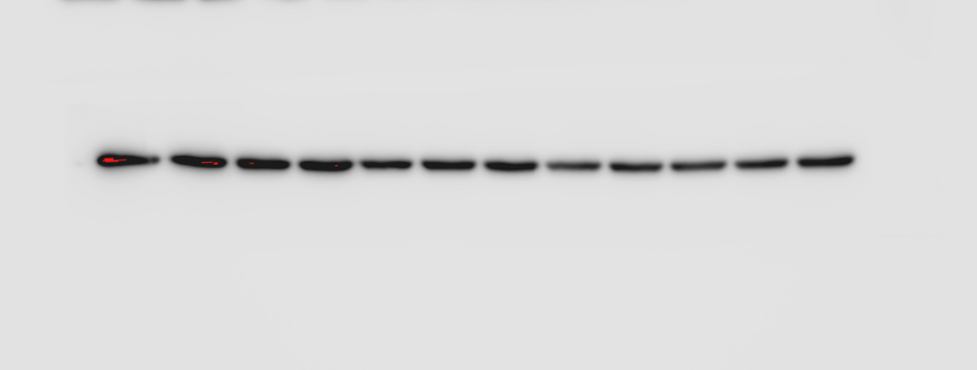

Supplement: Figure 6—source data 2. [file elife-85601-fig6-data2.zip › Figure 6 - Source Data 2/Original_pMLCquantificationFig6A_GAPDH_blot2.tif]
